# Phocoenamicins B and C, New Antibacterial Spirotetronates Isolated from a Marine *Micromonospora* sp.

**DOI:** 10.3390/md16030095

**Published:** 2018-03-16

**Authors:** Mercedes Pérez-Bonilla, Daniel Oves-Costales, Mercedes de la Cruz, Maria Kokkini, Jesús Martín, Francisca Vicente, Olga Genilloud, Fernando Reyes

**Affiliations:** Fundación MEDINA, Centro de Excelencia en Investigación de Medicamentos Innovadores en Andalucía, Parque Tecnológico Ciencias de la Salud, Avda. del Conocimiento 34, 18016 Armilla, Granada, Spain; daniel.oves@medinaandalucia.es (D.O.-C.); mercedes.delacruz@medinaandalucia.es (M.d.l.C.); maria.kokkini@medinandalucia.es (M.K.); jesus.martin@medinaandalucia.es (J.M.); francisca.vicente@medinaandalucia.es (F.V.); olga.genilloud@medinaandalucia.es (O.G.)

**Keywords:** spirotetronates, antimicrobial activity, *Micromonospora* sp., structural elucidation

## Abstract

Phocoenamicins B and C (**1** and **2**), together with the known spirotetronate phocoenamicin (**3**), were isolated from cultures of *Micromonospora* sp. The acetone extract from a culture of this strain, isolated from marine sediments collected in the Canary Islands, displayed activity against methicillin-resistant *Staphylococcus aureus* (MRSA), *Mycobacterium tuberculosis* H37Ra and *Mycobacterium bovis*. Bioassay-guided fractionation of this extract using SP207ss column chromatography and preparative reversed-phased HPLC led to the isolation of the new compounds **1** and **2** belonging to the spirotetronate class of polyketides. Their structures were determined using a combination of HRMS, 1D and 2D NMR experiments and comparison with the spectra reported for phocoenamicin. Antibacterial activity tests of the pure compounds against these pathogens revealed minimal inhibitory concentration (MIC) values ranging from 4 to 64 µg/mL for MRSA, and 16 to 32 µg/mL for *M. tuberculosis* H37Ra, with no significant activity found against *M. bovis* and vancomycin-resistant *Enterococcus faecium* (VRE) at concentrations below 128 µg/mL, and weak activity detected against *Bacillus subtilis* grown on agar plates.

## 1. Introduction

Antibiotics are compounds mainly produced by microorganisms that specifically kill or inhibit the growth of other microorganisms. Many antibiotics in today’s clinical use are secondary metabolites from actinomycetes or their semisynthetic derivatives [1]. Antibiotic resistance is rising to dangerously high levels all over the world. New resistance mechanisms are emerging and spreading globally, threatening our ability to treat common infectious diseases. Antibiotic resistance is accelerated by the misuse and overuse of antibiotics, as well as poor infection prevention and control. Those microorganisms that develop antimicrobial resistance are sometimes referred to as “superbugs” [2].

According to the World Health Organization (WHO) antimicrobial resistance (AMR) threatens the effective prevention and treatment of an ever-increasing range of infections caused by bacteria, parasites, viruses and fungi. WHO estimated that, in 2014, about 480,000 people developed multidrug-resistant tuberculosis (MDR-TB). Extensively drug-resistant tuberculosis (XDR-TB), a form of tuberculosis that is resistant to at least 4 of the core anti-TB drugs, has been identified in 105 countries [2]. Thus, bacterial infections represent an area of acute unmet medical needs and may become a deadly threat to mankind. Consequently, greater innovation and investment are required in research and development of new antimicrobial drugs, vaccines and diagnostic tools.

Nature is historically the source of new molecules exhibiting antibacterial activities; indeed, a considerable number of drugs have been developed from microorganisms of varied environments [3,4]. For several decades, the marine environment has been drawing attention, because it presents an enormous potential for the discovery of new therapeutic leads in the development of drugs to fight the current antibiotic resistance threats [5,6,7,8,9].

Marine actinobacteria are a great source of natural products with different biological properties including antimicrobial, anticancer, antiviral, insecticidal and enzyme inhibitory activities. They have attracted global interest in the last ten years for their ability to produce pharmacologically active compounds [1,9]. Actinomycetes are the most important biofactories for the production of therapeutic secondary metabolites, and most antibiotic discovery research focuses on the isolation of novel secondary metabolites from actinomycetes. The genus *Streptomyces* is the most prolific producer, with a 74% share of the natural products isolated from Actinomycetales, while non-*Streptomyces* species, also called rare actinomycetes, represent 26%. Rare or unusual actinomycetes have the potential to produce diverse, unique, unprecedented and occasionally structurally complicated compounds with excellent antibacterial potency and usually low toxicity [3].

Among the rare actinomycetes group, *Micromonospora* is one of the most fruitful genera, with more than 740 bioactive microbial metabolites discovered so far [3]. After the discovery of gentamicin [10], the genus *Micromonospora* became an important source in the drug discovery process. In general, soil and sediments are the main source for the isolation of antibiotic-producing *Micromonospora* strains. They also have been found to be constituents of endophytic actinobacterial populations recovered from plant tissues. *Micromonospora* species have also been isolated from water samples, sediments, and aquatic organisms such as ascidians, sponges, soft corals and mollusks [11].

To date, more than 70 spirotetronates have been reported from actinomycetes. These can be classified into three subgroups based on the size of the central ring, small (C_11_), medium (C_13_) and large (>C_13_) [12,13]. Among them, the spirotetronates maklamicin [14], nomimicin [15], together with PA-46101 A and B [16] and the recently isolated phocoenamicin [17] are representatives of the small size spirotetronate class.

During a screening in the search for novel antibiotic-producing microorganisms, we detected antimicrobial activity against the pathogens MRSA, *M. tuberculosis* H37Ra and *M. bovis* from the acetone extract of *Micromonospora* sp. CA-214671, collected in the Canary Islands. The LC–HRMS profile of this extract revealed the presence of potentially new secondary metabolites, which prompted a more detailed investigation of the extract. Extraction of the culture broth followed by a bioassay-guided fractionation of the extract led to the isolation of two new compounds (**1** and **2**), together with the recently reported phocoenamicin (**3**) [17], all of which belonged to the spirotetronate class of polyketides.

## 2. Results and Discussion

### 2.1. Isolation and Taxonomy of the Producing Microorganism

**Figure 1:**
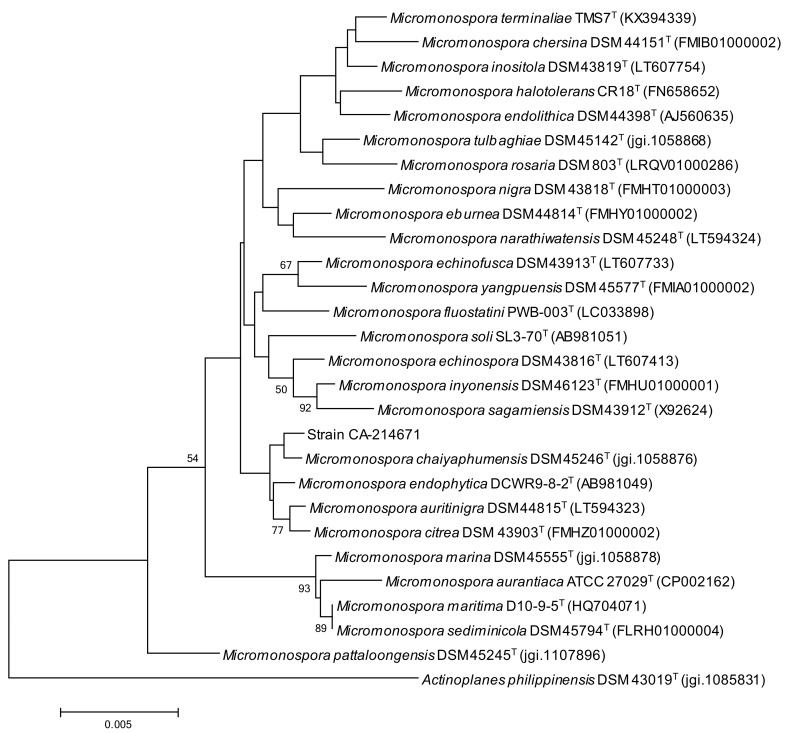
NJ tree built with Mega 6.06 based on 16S rRNA gene sequences of CA-214671 and its closest type strains of the genus *Micromonospora*. *Actinoplanes philippinensis* DSM 43019(T) was included as out-group. The scale bar indicates 0.005 substitutions per nucleotide. Numbers at the nodes indicate bootstrap support (%) based on NJ analysis of 1000 replicates (only values ≥ 50 are shown).

The strain CA-214671 was isolated from a marine cave sediment in Gran Canaria (Spain). A BLASTN search in the Eztaxon database of the PCR-amplified 16S rRNA nucleotide sequence (1290 nt) indicated that the strain is closely related to *Micromonospora chaiyaphumensis* (99.84 similarity) [18]. A phylogenetic tree was built employing the neighbor-joining (NJ) method corrected with the Jukes–Cantor algorithm [19,20] (Figure 1), which shows the close relationship of strain CA-214671 with *M. chaiyaphumensis* DSM 45246(T), although this association is not completely supported by the bootstrap value. Further multilocus sequence analysis (MLSA) phylogenetic studies are needed to confirm the species assignment of the *Micromonospora* strain CA-214671.

### 2.2. Extraction, Bioassay-Guided Isolation and Structural Elucidation

**Figure 2:**
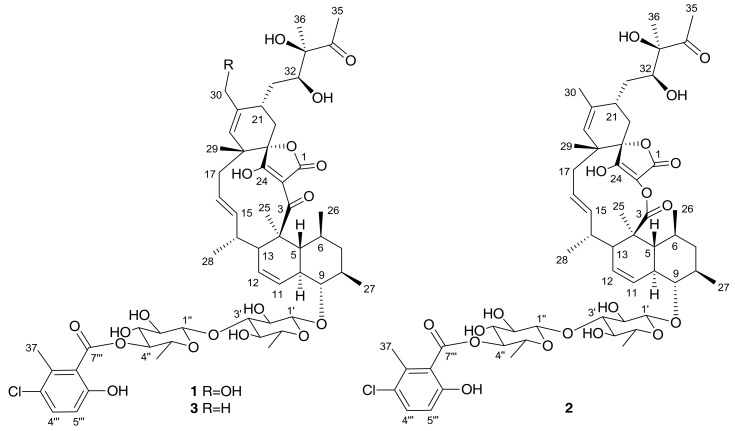
Structures of phocoenamicins isolated from the *Micromonospora* sp. (CA-214671) culture.

The producing strain was fermented in 3 L of FR23 medium supplemented with sea salts during 14 days at 28 °C. The fermentation broth was extracted with the same volume of acetone, followed by centrifugation, filtration and evaporation of the organic solvent. The remaining aqueous layer was subjected to bioassay-guided fractionation using SP207ss column chromatography and preparative reversed-phase HPLC, which led to the isolation of two new compounds, phocoenamicins B and C (**1** and **2**) together with the known spirotetronate phocoenamicin (**3**), recently reported as a potent *Clostridium difficile* inhibitor also isolated from a *Micromonospora* strain (Figure 2).

Compound **1** was obtained as an optically active white amorphous solid (${\left[\alpha \right]}_{D}^{25}$ – 16.7, MeOH). The (+)-ESI-TOF analysis showed an ammonium adduct at *m*/*z* 1104.4936 [M + NH_4_]^+^, from which a molecular formula of C_56_H_75_ClO_19_ was deduced for the compound. The IR spectrum showed characteristic absorptions of hydroxyl (3402 cm^−1^), carbonyl (1713 cm^−1^), and olefinic groups (1619, 1446 cm^−1^). Compound **1** displayed similar ^1^H and ^13^C NMR data to those recently described for phocoenamicin (**3**) [17]. The major difference between the ^1^H NMR spectra of both compounds was the presence of two oxygenated methylene protons at δ_H_ 3.98 and 4.14 in **1** (Table 1) instead of the methyl group at δ_H_ 1.74 ppm (Table S4 in Supplementary Materials) in phocoenamicin (**3**). The ^1^H NMR data, together with the HSQC spectrum, revealed that **1** contains a 1,2,3,4-tetrasubstituted benzene ring and two deoxysugar units, in addition to hydroxylated methines, olefinic protons and methyl groups (Table 1).

The main difference between the ^13^C NMR spectra of **1** and **3** was the presence of an oxygenated methylene carbon at δ_C_ 65.4 (Table 1) in **1**, in contrast to the methyl group at δ_C_ 22.3 observed for phocoenamicin (**3**) (Table S4 in Supplementary Materials). Other signals present in the ^13^C NMR spectrum of **1** accounted for the presence of two esters at δc 169.3 and 178.1 and two ketones at δ_C_ 201.3 and 215.3, two non-protonated carbons (δ_C_ 155.3 and 204.4), together with methine and quaternary sp^2^ carbons, quaternary sp^3^ carbons, methylene sp^3^, methine sp^3^, and methyl groups (Table 1). The distinctive resonances at δ_C_ 204.4, 201.3, 178.1 and 107.4 in the ^13^C NMR spectrum were characteristic for tetronic acid carbons [14].

**Figure 3:**
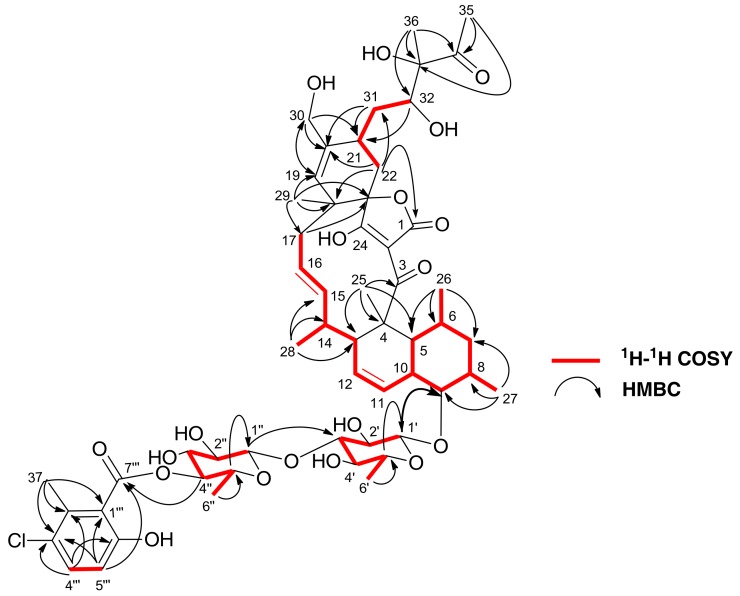
^1^H-^1^H COSY and key HMBC correlations for **1**.

The full planar structure of **1** was assigned based on its 1D and 2D NMR spectroscopic data. The ^1^H-^1^H COSY spectrum of **1** displayed a series of correlations establishing the presence of four main fragments (Figure 3), similar to those found in the structure of **3**. Correlations in the HMBC spectrum allowed the connection of the ^1^H-^1^H COSY-defined fragments as shown in Figure 3 and confirmed a carbon skeleton identical to that of phocoenamicin (**3**). The new oxygenated methylene group with respect to the structure of phocoenamicin was located at C-30 based on HMBC correlations from both H_2_-30 to C-19, C-20 and C-21, and from H-19 to C-30. Fragments related to the glycosyl moieties and their linkage positions were deduced based on the HMBC cross-peaks between the anomeric proton H-1′ of the first unit of 6-d-deoxyglucose and the oxygenated carbon C-9 of the decalin ring system. The long-range correlation from the anomeric proton H-1″ to C-3′ revealed that C-1″ of the second 6-deoxyglucose unit is attached to C-3′ of the first unit through an oxygen bridge linkage. Finally, a 3-chloro-6-hydroxy-2-methylbenzoate moiety, identical to that found in phocoenamicin [17] and similar to that of 2′′′-hydroxychlorothricin [21] was also present in the structure. HMBC correlation from H-4″ to C-7′′′ indicated that this aromatic ring was connected through an ester linkage to 4″ of the second 6-deoxyglucose unit.

**Figure 4:**
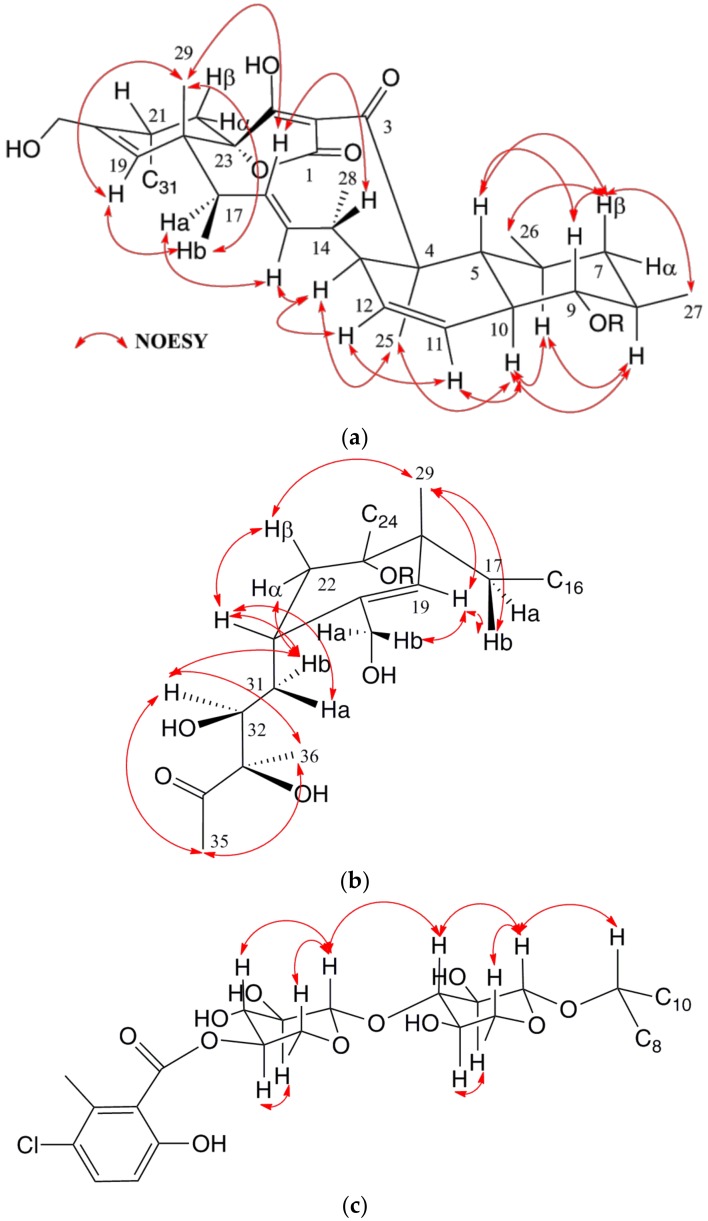
Conformation and configuration of the decalin (**a**), cyclohexene (**b**) and sugars moieties (**c**) of **1** determined by NOESY data and *J*-based analysis.

The relative configuration of **1** was established based on NOESY experiments (Figure 4a–c). Correlations observed around the decalin moiety indicated 1,3-diaxial relationships for H-5, H-7β and H-9, as well as for H-6, H-8 and H-10, a *trans* ring fusion of this decalin unit, and the equatorial orientation of the methyl groups at C-6 and C-8 (Figure 4a). A zig-zag conformation was suggested for the C-13 to C-18 chain based on the NOEs, along with the large *^3^J_HH_* coupling constants of 15.2 and 11.8 Hz between H-15/H-16 and H-16/H-17a, respectively, which in turn defined H-13, H-15 and H_3_-25 to be in the bottom face of the molecule (Figure 4a). Additional NOESY correlations (Figure 4b) placed H_3_-29, H-22β and H-21 on the top face of the cyclohexene ring present in the molecule and confirmed its chair conformation. Conformation of the side chain at C-21 was deduced from the NOESY correlations for H-22α/H-31b, H-31b/H-32 and H-32/H_3_-36 setting an *anti*-relationship between H-31a and H-32, and between H-32 and the hydroxy group at C-33, which in turn indicated a configuration S*/R* at C-32 and C-33 carbons, respectively (Figure 4b), the same configuration recently described for phocoenamicin [17]. The configuration at the spirocarbon C-23 could not be determined experimentally due to the overlapped signals, so it was hypothesized that the tetronic acid stereogenic center should be assigned a S* configuration in alignment with other spirotetronates [14,17].

Finally, the relative configurations of the sugars were determined by NOESY correlations (Figure 4c), establishing the axial orientation of all protons and confirming two units of β-6-deoxyglucopyranoside whose absolute configuration is proposed as D based on that previously determined for the related compound phocoenamicin [17]. These data confirmed the structure of compound **1** as a new member of the spirotetronate family comprising a spiro-tetronic acid motif embedded within an eleven-membered macrocyclic core linked to a cyclohexene ring and to a *trans*-decalin moiety. Compound **1** also contains a side chain at C-21 and a disaccharide connected to a substituted benzoic acid moiety with 23 chiral centers in its structure. NOESY correlations confirmed the same configuration of the chiral centers of our molecule with respect to those proposed for phocoenamicin, and the isolation of the latter compound from our culture broth was also in favor of this stereochemical proposal. Therefore, the structure of compound **1** was confirmed as 30-hydroxyphocoenamicin, for which we propose the name phocoenamicin B.

Compound **2** was obtained as an optically active white amorphous solid (${\left[\alpha \right]}_{D}^{25}$ + 24.3, MeOH). The (+)-HRESIMS data showed an adduct ion at *m*/*z* 1104.4929 [M + NH_4_]^+^, accounting for a molecular formula C_56_H_75_ClO_19_, isomeric with that of **1**. The IR spectrum showed characteristic absorptions of hydroxyl (3425 cm^−1^), carbonyl (1713 cm^−1^), and olefinic groups (1590, 1446 cm^−1^). ^1^H and ^13^C chemical shifts of **2** were similar to those of **1** for the *trans*-decalin, the disaccharide, and the benzoate moiety. The major difference between the ^1^H NMR data of both compounds was the presence of a methyl group at δ_H_ 1.73 instead of the two methylene protons at δ_H_ 3.98 and 4.14 (Table 1). Additional minor chemical shift differences were observed for most of the protons present in the molecule (Table 1).

The major differences in the ^13^C NMR spectra were the presence of a methyl group at δ_C_ 22.4 and the lack of the methylene group at δ_C_ 65.4 in **2** with respect to **1**. Additionally, instead of a ketone carbonyl signal of C-3, a signal of an ester carbonyl carbon was observed at δ_C_ 176.2 which was supported by the HMBC correlation from H_3_-25 and this carbon (Figure S1). HMBC correlations were observed from the methyl protons at δ_H_ 1.73 to the carbons at δ_C_ 133.5 (C-19) and 134.8 (C-20) indicating that the methyl group was attached to the cyclohexene ring at C-20 (Supplementary Information Figure S1). The relative configurations of **2** were established based on NOESY experiments (Supplementary Information Figure S2). The ^1^H and ^13^C chemical shifts observed in **2** suggested the presence of a 12-membered core macrocycle, consisting of a macrolactone ring closely related to that found in chlorothricins and PA-46101 A and B antibiotics [12,16,21]. Accordingly, **2** was identified as a macrolactone core ring, spiro-tetronic acid derivative linked to a cyclohexene ring and to a *trans*-decalin moiety, which was named phocoenamicin C. Compound **2** most probably derives from the enzymatic Baeyer–Villiger oxidation of phocoenamicin (**3**). Similar oxidative biotransformations have also been described in the biosynthesis of mithramycin [22].

Compound **3** was obtained as an optically active white amorphous solid (${\left[\alpha \right]}_{D}^{25}$ – 5.9, MeOH). The (+)-HRESIMS showed an adduct ion at *m*/*z* 1088.4979 [M + NH_4_]^+^, from which the molecular formula C_56_H_75_ClO_18_ was deduced. Its NMR data (Table S4 in Supplementary Materials) were similar to those of **1** and **2** and, although recorded in a different solvent, were in good agreement with those previously published for phocoenamicin [17]. 

The isolated compounds share a small macrocycle framework, like the aforementioned spirotetronates maklamicin [14] and nomimicin [15], together with PA-46101 A and B (whose aglycone structure shares important features with the tetronate antibiotic chlorothricin) [16]. Unique structural features of the two new compounds herein described are the presence of two 6-deoxyglucose moieties similar to that observed in 2′′′-hydroxychlorothricin [21] together with the diol side chain detected so far only in phocoenamicin [17].

### 2.3. Antimicrobial Activity

All the isolated compounds were evaluated for their antibacterial activity against several Gram-positive bacteria, methicillin-resistant *S. aureus* (MRSA) MB5393, *M. tuberculosis* (H37Ra) ATCC 25177, *M. bovis* ATCC 35734, vancomycin-resistant *E. faecium* (VRE) MB5571 and *B. subtilis* MB964 (Table 2). The results showed that **1** and **3** exhibited a significant activity against MRSA, with minimum inhibitory concentration (MIC) values ranging from 4 to 8 µg/mL to 8 to 16 µg/mL for **3** and **1**, respectively. Compound **2** was significantly less active, inhibiting the growth of MRSA (MIC 32–64 µg/mL). In contrast, moderate to poor activity was observed against both *Mycobacterium* strains. Compound **3** displayed the highest inhibitory effect against *M. tuberculosis* H37Ra with a MIC value ranging from 16 to 32 µg/mL. On the contrary, negligible activity was observed against *M. bovis* for all compounds. Only **3** displayed weak activity against vancomycin-resistant *E. faecium*, with a MIC value of 32–64 µg/mL whereas phocoenamicin B (**1**) showed the best inhibitory activity against *B. subtilis* in agar plates with a diameter of the zone of inhibition of 7 mm when 2 μg of the compound was applied to the agar surface.

Based on the structure–activity relationship findings, similarly to other related spirotetronates, phocoenamicins B and C showed antibacterial activity against Gram-positive bacteria. Although phocoenamicin C possess a similar structure to that of phocoenamicin, they showed different biological activities that are likely due to the macrocycle core bearing a carboxylic ester instead of a ketone group, possibly the requirement for the activity. Findings related to the structure–activity relationships in other small spirotetronates revealed that conformational rigidity plays a key role in achieving the proper orientation of the enone moiety for interaction with the target [12].

## 3. Materials and Methods

### 3.1. General Experimental Procedures

Optical rotations were measured using a Jasco P-2000 polarimeter (JASCO Corporation, Tokyo, Japan). UV spectra were obtained with an Agilent 1100 DAD (Agilent Technologies, Santa Clara, CA, USA). IR spectra were recorded on a JASCO FT/IR-4100 spectrometer (JASCO Corporation, Tokyo, Japan) equipped with a PIKE MIRacle^TM^ single reflection ATR accessory (PIKE Thecnologies Inc., Madison, WI, USA). NMR spectra were recorded on a Bruker Avance III spectrometer (500 and 125 MHz for ^1^H and ^13^C NMR, respectively) equipped with a 1.7 mm TCI MicroCryoProbe^TM^ (Bruker Biospin, Falländen, Switzerland). Chemical shifts were reported in ppm using the signals of the residual solvent as internal reference (δ_H_ 3.31 and δ_C_ 49.15 for CD_3_OD). LC–MS and LC–HRMS analyses were performed as described previously [23].

### 3.2. Taxonomical Identification of the Producing Microorganism

The 16S rRNA gene was PCR-amplified and sequenced as previously described [24]. The resulting DNA sequence lectures were aligned and visually inspected with Bionumerics 6.6 (Applied Maths, Sint-Martens-Latem, Belgium) to obtain the 16S rRNA sequence (1290 nt) (Supplementary Information Text S3).

### 3.3. Fermentation of the Producing Microorganism

A 3 L fermentation of the producing microorganism was generated as follows: a fresh seed culture of *Micromonospora* sp. CA-214671 was obtained inoculating a 25 × 150 mm tube containing 16 mL of ATCC-2-M medium (soluble starch 20 g/L, glucose 10 g/L, NZ Amine Type E 5 g/L, meat extract 3 g/L, peptone 5 g/L, yeast extract 5 g/L, sea salts 30 g/L, calcium carbonate 1 g/L, pH 7) with a freshly thawed inoculum stock of the strain. The tube was incubated in an orbital shaker at 28 °C, 220 rpm, 70% relative humidity for 7 days. The grown culture was then used to inoculate two 250 mL flasks each containing 50 mL of ATCC-2-M medium (5% *v*/*v*). The flasks were incubated in an orbital shaker at 28 °C, 220 rpm and 70% humidity for 7 days. The content of both flasks was then mixed, and the mixture was used to inoculate sixty 250 mL flasks each containing 50 mL of FR23 fermentation medium supplemented with sea salts (glucose 5 g/L, soluble starch from potato 30 g/L, cane molasses 20 g/L, cottonseed flour 20 g/L, sea salts 30 g/L, pH 7) (2.5% *v*/*v*). The flasks were incubated in an orbital shaker at 28 °C, 220 rpm and 70% humidity during 14 days before harvesting.

### 3.4. Extraction and Bioassay-Guided Isolation

After 14 days of fermentation, 50 mL of acetone was added to each Erlenmeyer flask (60 Erlenmeyer flasks which were shaken for 1 h in a Kühner at 200 rpm and 25 °C). Afterwards, the mixture was centrifuged at 8500 rpm for 10 min, and the supernatant was separated from the mycelium by decantation. Supernatant was then filtered under vacuum and the extract was concentrated in a rotary evaporator to remove the acetone. The aqueous residue was loaded onto a SP207ss resin column (65 g, 32 × 100 mm) and eluted with an acetone/H_2_O stepped gradient (10/90 for 7.2 min, 20/80 for 7.2 min, 40/60 for 7.1 min, 60/40 for 7.1 min, 80/20 for 6 min and 100/0 for 14.3 min, 10 mL/min, 20 mL/fraction) to give twenty-five fractions. Fractions containing the compounds of interest from this chromatography (19 to 22) were pooled and subjected to preparative reversed-phase HPLC (Waters™ XBridge^®^ Prep C_18_ (Waters Corporation, Milford, MA, USA), 19 × 250 mm, 5 µm, 14 mL/min, UV detection at 210 and 280 nm, 8.75 mL/fraction) using H_2_O as solvent A and CH_3_CN as solvent B. Elution was carried out using isocratic conditions of 5% B for 5 min and then a linear gradient from 5% to 100% B for 52 min, held at 100% B for 5 min, yielding 84 fractions that were tested against MRSA, *M. tuberculosis* H37Ra and *M. bovis*.

Subfractions 59–76 were active against MRSA, *M. tuberculosis* H37Ra and *M. bovis*. Subfractions 59 to 61 were further purified by semi-preparative reversed-phase HPLC (Waters™ XBridge^®^ Semiprep C_18_, 10 × 150 mm, 5 µm, 3.8 mL/min, UV detection at 210 and 230 nm, 1.82 mL/fraction) using H_2_O as solvent A and CH_3_CN as solvent B. Elution was carried out using isocratic conditions of 40% B for 5 min, linear gradient from 40% B to 100% B in 35 min to yield **1** (t_R_ 22 min, 0.8 mg).

Subfractions 68 and 69 were further purified by semi-preparative reversed-phase HPLC (Waters™ XBridge^®^ Semiprep C_18_, 10 × 150 mm, 5 µm, 3.8 mL/min, UV detection at 210 and 230 nm, 1.82 mL/fraction) using the same chromatographic conditions to yield **2** (t_R_ 26 min, 1.2 mg).

Subfractions 74 to 76 were further purified by semi-preparative reversed-phase HPLC (Waters™ XBridge^®^ Semiprep C_18_, 10 × 150 mm, 5 µm, 3.8 mL/min, UV detection at 210 and 230 nm, 1.82 mL/fraction) using the same chromatographic conditions to yield **3** (t_R_ 31 min, 1.3 mg).

Phocoenamicin B (**1**): white amorphous solid; ${\left[\alpha \right]}_{D}^{25}$ – 16.7 (*c* 0.16, MeOH); UV (DAD) λ_max_ 230, sh 290, 320 nm; IR (ATR) ν_max_ 3402, 2924, 1713, 1619, 1446, 1393, 1068, 1024 cm^−1^; for ^1^H and ^13^C NMR data see Table 1; (+)-HRESIMS *m*/*z* 1104.4936 [M + NH_4_]^+^ (calcd for C_56_H_79_ClNO_19_^+^, 1104.4929).

Phocoenamicin C (**2**): white amorphous solid; ${\left[\alpha \right]}_{D}^{25}$ + 24.3 (*c* 0.17, MeOH); UV (DAD) λ_max_ sh 230, sh 250, 290 nm; IR (ATR) ν_max_ 3425, 2926, 1713, 1590, 1446, 1378, 1070, 1026 cm^−1^; for ^1^H and ^13^C NMR data see Table 1; (+)-HRESIMS *m*/*z* 1104.4929 [M + NH_4_]^+^ (calcd for C_56_H_79_ClNO_19_^+^, 1104.4929).

Phocoenamicin (**3**): white amorphous solid; ${\left[\alpha \right]}_{D}^{25}$ – 5.9 (*c* 0.35, MeOH); UV (DAD) λ_max_ 230, sh 290, 320 nm; IR (ATR) ν_max_ 3404, 2925, 1714, 1618, 1446, 1391, 1295, 1069, 1023 cm^−1^; for ^1^H and ^13^C NMR data see Table S4 Supplementary information; (+)-HRESIMS *m*/*z* 1088.4979 [M + NH_4_]^+^ (calcd for C_56_H_79_ClNO_18_^+^, 1088.4980).

### 3.5. Antibacterial Activity Assay

The antibacterial activity of the isolated compounds was evaluated using sequential 2-fold serial dilutions of each compound in DMSO to provide 10 concentrations starting at 128 µg/mL for all the assays. Three Gram-positive bacteria including methicillin-resistant *S. aureus* (MRSA) MB5393, *M. tuberculosis* (H37Ra) ATCC 25177, *M. bovis* ATCC 35734, vancomycin-resistant *E. faecium* MB5571 and *B. subtilis* MB964 were used in this study, following previously described methodologies [25,26,27].

## 4. Conclusions

Two new spirotetronates have been isolated from the marine actinomycete *Micromonospora* sp. The new structures comprise a spiro-tetronic acid motif embedded within an eleven-membered macrocyclic core linked to a cyclohexene ring and to a *trans*-decalin moiety. Unique structural features of the two new compounds are the presence of two 6-d-deoxyglucose moieties together with the diol side chain only detected in phocoenamicin to date [17].

Concerning their biological activity, phocoenamicins B (**1**) and C (**2**) showed antibacterial activity against Gram-positive bacteria. Compounds **1** and **3** displayed significant activity against MRSA, one of the most common causes of hospital-acquired infections, while moderate to negligible activity was observed against *M. tuberculosis* H37Ra, *M. bovis*, *B. subtilis* and *E. faecium* VRE. Additionally, moderate anti-tuberculosis activity against *M. tuberculosis* was displayed by **2** and **3**. Apparently, conformational rigidity to achieve the proper orientation of the enone moiety could play a key role in the interaction with the target, revealing the possible structure–activity relationship for these compounds.

Our study also confirms that *Micromonospora* species producing spirotetronates are widely spread within the marine environment, and current studies by LC–MS of our strain collection point out that other *Micromonospora* strains producers of phocoenamicins have also been isolated from terrestrial environments. Furthermore, the present study highlights that the marine environment offers an enormous potential for the discovery of compounds with new chemical scaffolds, thus representing a great opportunity to find new antibiotics to confront multi-resistant bacteria. The challenge in the future will be the discovery of more chemically and hopefully mechanistically new agents to satisfy these needs.

## Figures and Tables

**Table 1:** NMR spectroscopic data (500 MHz, CD_3_OD) for phocoenamicins B (**1**) and C (**2**).

Position	Phocoenamicin B (1)		Phocoenamicin C (2)	
	δ_C_, Type	δ_H_, Mult. (*J* in Hz)	δ_C_, Type	δ_H_, Mult. (*J* in Hz)
1	178.1, C ^a^		172.7, C ^a^	
2	107.4, C		n.d *	
3	201.3, C ^a^		176.2, C	
4	51.2, C		47.1, C	
5	43.9, CH	1.85, m	44.1, CH	1.60, m
6	39.6, CH	1.42, m	38.6, CH	1.52, m
7α	46.0, CH_2_	1.70, m	46.0, CH_2_	1.75, m
7β		1.20, m		1.11, m
8	40.9, CH	1.60, m	40.8, CH	1.63, m
9	88.9, CH	3.03, t (9.2)	88.0, CH	3.03, t (9.8)
10	48.4, CH	1.93, m	48.3, CH	1.92, m
11	126.4, CH	6.22, d (10.4)	126.4, CH	6.27, dd (10.3, 2.5)
12	127.9, CH	5.55, dd (9.8, 5.9)	128.2, CH	5.63, ddd (10.3, 6.2, 2.4)
13	42.1, CH	2.72, m	50.6, CH	2.00, m
14	39.8, CH	2.10, m	40.7, CH	2.21, m
15	141.5, CH	5.27, m	136.3, CH	4.90, m
16	123.7, CH	5.18, dd (15.2, 11.8)	129.3, CH	5.27, ddd (15.7, 11.6, 3.1)
17a	44.1, CH_2_	2.34, m	43.1, CH_2_	2.32, m
17b		1.85, m		1.70, m
18	41.1, C		44.3, C	
19	133.6, CH	5.26, s	133.5, CH	4.99, brs
20	138.0, C		134.8, C	
21	30.1, CH	2.58, m	34.6, CH	2.33, m
22α	30.5, CH_2_	1.68, m	29.8, CH_2_	1.73, m
22β		2.26, m		2.38, m
23	87.1, C		87.0, C	
24	204.4, C ^a^		n.d *	
25	16.8, CH_3_	1.53, s	17.3, CH_3_	1.31, s
26	24.0, CH_3_	0.80, brs	22.1, CH_3_	0.93, d (6.8)
27	20.2, CH_3_	1.02, d (6.2)	20.0, CH_3_	1.02, d (6.4)
28	21.5, CH_3_	0.80, brs	22.5, CH_3_	0.89, d (7.2)
29	24.6, CH_3_	1.23, brs	23.7, CH_3_	1.26, s
30a	65.4, CH_2_	4.14, d (13.2)	22.4, CH_3_	1.73, s
30b		3.98, d (13.2)		
31a	33.5, CH_2_	2.07, m	33.9, CH_2_	1.94, m
31b		1.69, m		1.73, m
32	74.1, CH	3.86, dd (11.2, 1.4)	74.1, CH	3.84, dd (10.9, 1.9)
33	83.5, C		83.5, C	
34	215.3, C		215.2, C	
35	25.7, CH_3_	2.24, s	25.6, CH_3_	2.23, s
36	22.2, CH_3_	1.24, s	22.1, CH_3_	1.20, s
1′	104.0, CH	4.36, d (7.3)	104.0, CH	4.35, d (7.3)
2′	75.4, CH	3.45, m	75.4, CH	3.45, m
3′	88.6, CH	3.46, m	88.6, CH	3.48, m
4′	75.7, CH	3.11, t (8.8)	75.6, CH	3.11, t (8.8)
5′	72.8, CH	3.22, m	72.9, CH	3.24, m
6′	18.3, CH_3_	1.27, d (6.0)	18.3, CH_3_	1.28, d (6.0)
1″	105.4, CH	4.61, d (7.8)	105.4, CH	4.61, d (7.9)
2″	76.1, CH	3.42, t (8.5)	76.1, CH	3.42, t (8.6)
3″	75.3, CH	3.64, t (9.3)	75.4, CH	3.64, t (9.4)
4″	77.9, CH	4.89, **	77.9, CH	4.87, **
5″	71.7, CH	3.69, dq (9.7, 6.1)	71.7, CH	3.69, dq (9.7, 6.2)
6″	18.0, CH_3_	1.35, d (6.1)	18.0, CH_3_	1.35, d (6.2)
1′′′	124.3, C		124.3, C	
2′′′	135.6, C		135.6, C	
3′′′	126.0, C		126.0, C	
4′′′	132.4, CH	7.25, d (8.7)	132.4, CH	7.25, d (8.7)
5′′′	115.9, CH	6.70, d (8.7)	115.8, CH	6.70, d (8.7)
6′′′	155.3, C		155.3, C	
7′′′	169.3, C		169.3, C	
37	17.3, CH_3_	2.36, s	17.9, CH_3_	2.36, s

^a^ Assigned based on 2D correlations. * n.d. = not detected; ** Obscured by the water peak.

**Table 2:** Antimicrobial bioassay results of phocoenamicins **1**–**3**.

Compounds	MIC (μg/mL)				ZOI * mm(μg)
	*S. aureus* MB5393	*M. tuberculosis* ATCC 25177	*M. bovis* ATCC 35734	*E. faecium* MB5571	*B. subtilis* MB964
phocoenamicin B (1)	8–16	>128	>128	>128	7 (2)
phocoenamicin C (2)	32–64	32	>128	>128	7 (4)
phocoenamicin (3)	4–8	16–32	>128	32–64	7 (4)
vancomycin	2–4			>128	
streptomycin		1.6–3.2	0.4-0.8		
gentamicin					8 (0.25)
penicillin G					19 (0.06)

* ZOI = zone of inhibition.

## Supplementary Materials

The following are available online at www.mdpi.com/1660-3397/16/3/95/s1, Figure S1: ^1^H-^1^H COSY and key HMBC correlations for **2**; Figure S2: Conformation and configuration of the decalin, cyclohexene and sugars moieties of **2** determined by NOESY data and *J*-based analysis; Text S3: 16S rRNA gene sequence from strain CA-214671; Table S4: NMR spectroscopic data (500 Mz, CD_3_OD) for phocoenamicin (**3**); Figure S5: ^1^H-NMR (500 MHz, methanol-*d_4_*) spectrum of phocoenamicin B (**1**); Figure S6: ^13^C-NMR (125 MHz, methanol-*d_4_*) spectrum of phocoenamicin B (**1**); Figure S7: HSQC (methanol-*d_4_*) spectrum of phocoenamicin B (**1**); Figure S8: HMBC (methanol-*d_4_*) spectrum of phocoenamicin B (**1**); Figure S9: COSY (methanol-*d_4_*) spectrum of phocoenamicin B (**1**); Figure S10: NOESY (methanol-*d_4_*) spectrum of phocoenamicin B (**1**); Figure S11: TOCSY (methanol-*d_4_*) spectrum of phocoenamicin B (**1**); Figure S12: ^1^H-NMR (500 MHz, methanol-*d_4_*) spectrum of phocoenamicin C (**2**); Figure S13: ^13^C-NMR (125 MHz, methanol-*d_4_*) spectrum of phocoenamicin C (**2**); Figure S14: HSQC (methanol-*d_4_*) spectrum of phocoenamicin C (**2**); Figure S15: HMBC (methanol-*d_4_*) spectrum of phocoenamicin C (**2**); Figure S16: COSY (methanol-*d_4_*) spectrum of phocoenamicin C (**2**); Figure S17: NOESY (methanol-*d_4_*) spectrum of phocoenamicin C (**2**); Figure S18: TOCSY (methanol-*d_4_*) spectrum of phocoenamicin C (**2**).
