# Supplementary material for: Phocoenamicins B and C, New Antibacterial Spirotetronates Isolated from a Marine Micromonospora sp
Source: Mar Drugs. 2018 Mar 16;16(3):95. doi: 10.3390/md16030095 (PMC5867639; doi:10.3390/md16030095)
Supplement: Supplementary file 1 [file marinedrugs-16-00095-s001.pdf]

Supplementary Information for

Phocoenamicins B and C, new antibacterial spirotetronates from the Marine Actinomycete *Micromonospora chaiyaphumensis*

Mercedes Pérez-Bonilla*, Daniel Oves-Costales, Mercedes de la Cruz, Maria Kokkini, Jesús Martín, Francisca Vicente, Olga Genilloud and Fernando Reyes*

Fundación MEDINA, Centro de Excelencia en Investigación de Medicamentos Innovadores en Andalucía. Parque Tecnológico Ciencias de la Salud. Avda. del Conocimiento 34, 18016, Armilla, Granada, Spain; daniel.oves@medinaandalucia.es (D.O.-C.); mercedes.delacruz@medinaandalucia.es (M.d.l.C.); maria.kokkini@medinandalucia.es (M.K.); jesus.martin@medinaandalucia.es (J.M.); francisca.vicente@medinaandalucia.es (F.V.); olga.genilloud@medinaandalucia.es (O.G.)

* Correspondence: mercedes.perez@medinaandalucia.es (M.P.-B.); fernando.reyes@medinaandalucia.es (F.R.) Tel.: +34-958-993-965 (F.R.)

Contents:

Figure S1. ^{1}H-^{1}H COSY and key HMBC correlations for **2**.
Figure S2. Conformation and configuration of the decalin, cyclohexene and sugars moieties of **2** determined by NOESY data and *J*-based analysis.
Text S3. 16S rRNA gene sequence from strain CA-214671.
Table S4. NMR spectroscopic data (500 Mz, CD_3OD) for phocoenamicin (**3**).
Figure S5. ^{1}H-NMR (500 MHz, methanol-d_4) spectrum of phocoenamicin B (**1**).
Figure S6. ^{13}C-NMR (125 MHz, methanol-d_4) spectrum of phocoenamicin B (**1**).
Figure S7. HSQC (methanol-d_4) spectrum of phocoenamicin B (**1**).
Figure S8. HMBC (methanol-d_4) spectrum of phocoenamicin B (**1**).
Figure S9. COSY (methanol-d_4) spectrum of phocoenamicin B (**1**).
Figure S10. NOESY (methanol-d_4) spectrum of phocoenamicin B (**1**).
Figure S11. TOCSY (methanol-d_4) spectrum of phocoenamicin B (**1**).
Figure S12. ^{1}H-NMR (500 MHz, methanol-d_4) spectrum of phocoenamicin C (**2**).
Figure S13. ^{13}C-NMR (125 MHz, methanol-d_4) spectrum of phocoenamicin C (**2**).
Figure S14. HSQC (methanol-d_4) spectrum of phocoenamicin C (**2**).
Figure S15. HMBC (methanol-d_4) spectrum of phocoenamicin C (**2**).
Figure S16. COSY (methanol-d_4) spectrum of phocoenamicin C (**2**).
Figure S17. NOESY (methanol-d_4) spectrum of phocoenamicin C (**2**).
Figure S18. TOCSY (methanol-d_4) spectrum of phocoenamicin C (**2**).

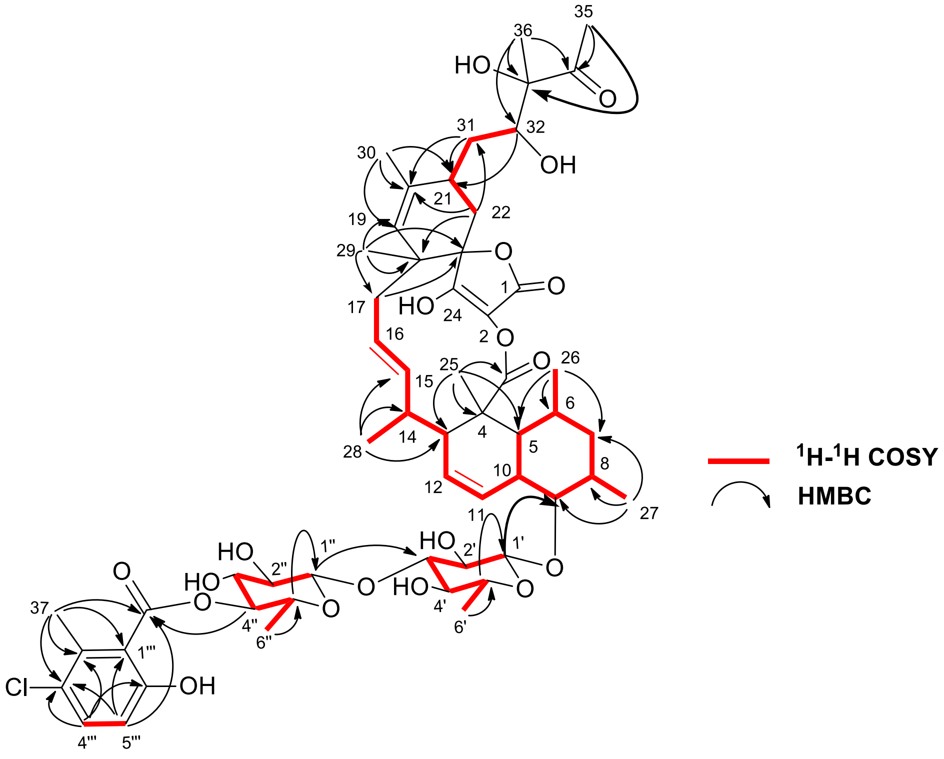

Figure S1. ¹H-¹H COSY and key HMBC correlations for **2**.

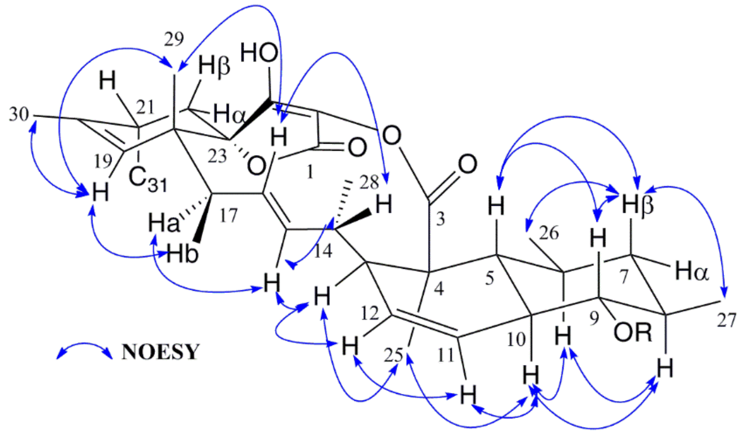

(S2.a)

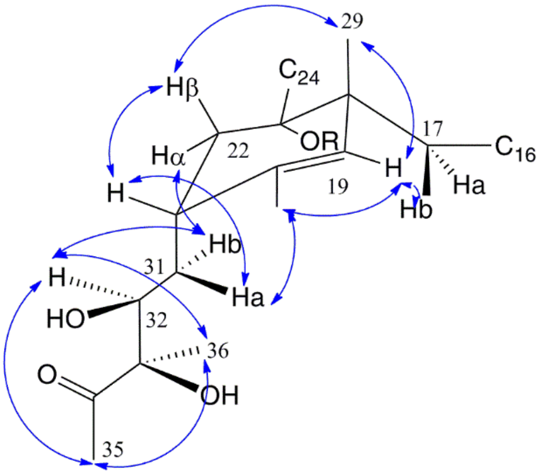

(S2.b)

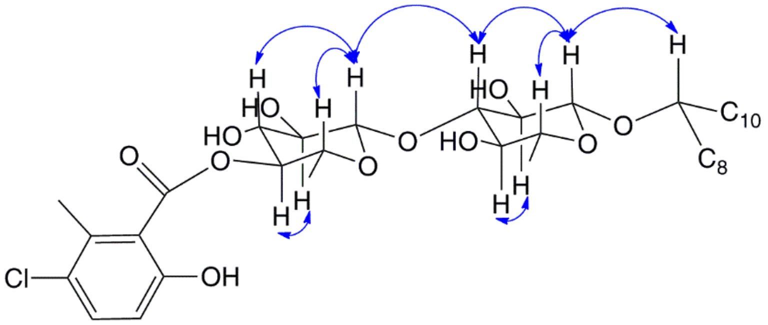

(S2.c)

Figure S2. Conformation and configuration of the decalin, cyclohexene and sugars moieties of **2** determined by NOESY data and *J*-based analysis.

Text S3. 16S rRNA gene sequence from strain CA-214671.

cgacttcgtcccaatcgccagccccaccttcgacggctccctccacaagggttgggccaccggcttcgggtgttgccgactttcgtgacgtgacg
ggcggtgtgtacaaggcccgggaacgtattcaccgcagcgttgctgatctgcgattactagcgactccgacttcacggggtcgagttgcagac
cccgatccgaactgagaccggctttttgggattcgctccacctcacggtatcgcagcccattgtaccggccattgtagcatgcgtgaagccctgg
acataaggggcatgatgacttgacgtcatccccaccttcctccgagttgaccccggcagtcttcgatgagtccccgccataacgcgctggcaaca
tcgaacgagggttgcgctcgttgcgggacttaacccaacatctcacgacacgagctgacgacagccatgcaccacctgtgaccgccc ccgaag
gacctsccatctctgncagttttgcggccatgtcaaacccaggtaaggttcttcgcgttgcatcgaattaatccgcatgctccgccgcttgtgcgg
gcccccgtcaattcctttgagttttagccttgcggccgtactccccaggcggggcgcttaatgcgttagctgcggcacagrgaaccggagaggc
cccccacacctagcgcccaacgtttacagcgtggactaccagggtatctaatcctgttcg ctccccacgctttcgctcctcagcgtcagtatcggcc
cagagacccgccttcgccaccggtgttcctcctgatatctgcgcatttcaccgctacaccaggaattccagtctcccctaccgaactctagcctgcc
cgtatcgactgcaggcccgcagttgagctgcgggttttcacagtcgacgcgacaagccgcctacgagctctttacgcccaataaatccggacaa
cgctcgcgccctacgtcttaccgcggctgctggcacgtagttggccggcgcttcttctgcaggtaccgtcacttacgcttcgtccctgctgaaaga
ggtttacaacccgaaggccgtcatccctcacgcggcgtcgctgcatcaggcttccgcccattgtgcaatattccccactgctgcctcccgtaggag
tctgggccgtgtctcagtcccagtgtggccggtcgccctctcaggccggctacccgtcgtcgccttggtaggccatcaccccaccaacaagc tga
taggccgcgagcccatcccaggccgaaaaactttccacccccaaccatgcggtc

Table S4. NMR spectroscopic data (500 Mz, CD_3OD) for phocoenamicin (**3**).

	phocoenamicin (**3**)	
**Position**	**δ_C, type**	**δ_H (*J* in Hz)**
1	177.8, C	
2	107.3, C	
3	200.1, C	
4	50.9, C	
5	43.6, CH	1.82, m
6	39.5, CH	1.49, m
7α	45.7, CH_2	1.72, m
7β		1.21, m
8	40.9, CH	1.64, m
9	88.6, CH	3.04, t (9.7)
10	48.4, CH	1.97, m
11	126.0, CH	6.29, d (10.0)
12	126.7, CH	5.57, dd (10.0, 6.2)
13	43.0, CH	2.67, *
14	40.0, CH	2.05, m
15	144.1, CH	5.36, dd (15.1, 9.1)
16	123.2, CH	5.14, dd (15.1, 10.4)
17a	44.0, CH_2	2.31, m
17b		1.86, m
18	41.2, C	
19	132.1, CH	5.02, s
20	134.6, C	
21	34.3, CH	2.37, m
22α	30.3, CH_2	2.28, m
22β		1.71, m
23	87.5, C	
24	205.7, C	
25	17.0, CH_3	1.59, s
26	23.7, CH_3	0.82, brs
27	20.1, CH_3	1.03, d (6.2)

28	21.5, CH_3	0.82, brs
29	24.7, CH_3	1.23, brs
30	22.3, CH_3	1.74, s
31a	33.7, CH_2	1.95, m
31b		1.74, m
32	74.0, CH	3.82, d (10.6)
33	83.4, C	
34	215.4, C	
35	25.7, CH_3	2.24, s
36	22.2, CH_3	1.22, s
1'	104.0, CH	4.35, d (6.2)
2'	75.3, CH	3.45, m
3'	88.6, CH	3.46, m
4'	75.6, CH	3.11, t (8.7)
5'	72.9, CH	3.22, m
6'	18.4, CH_3	1.27, d (6.0)
1''	105.4, CH	4.61, d (7.8)
2''	76.0, CH	3.42, t (8.6)
3''	75.3, CH	3.65, t (9.7)
4''	77.9, CH	4.89, *
5''	71.7, CH	3.68, m
6''	18.0, CH_3	1.36, d (6.2)
1'''	124.3, C	
2'''	135.6, C	
3'''	126.0, C	
4'''	132.4, CH	7.25, d (8.7)
5'''	115.8, CH	6.70, d (8.7)
6'''	155.3, C	
7'''	169.3, C	
37	17.9, CH_3	2.36, s

*Multiplicity not clear, the signal is overlapped.

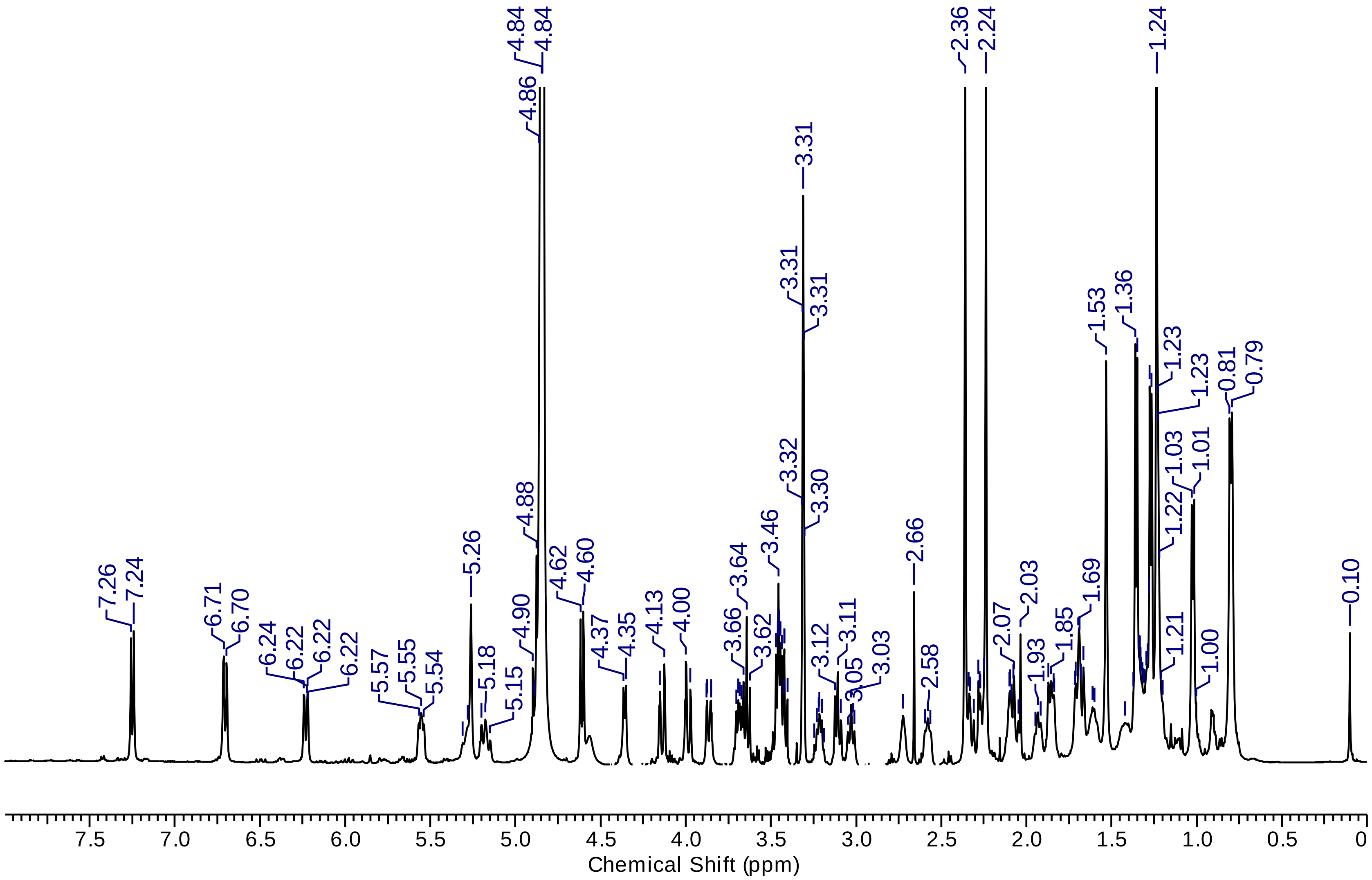

Figure S5. ^{1}H-NMR (500 MHz, methanol-d_4) spectrum of phocoenamicin B (**1**).

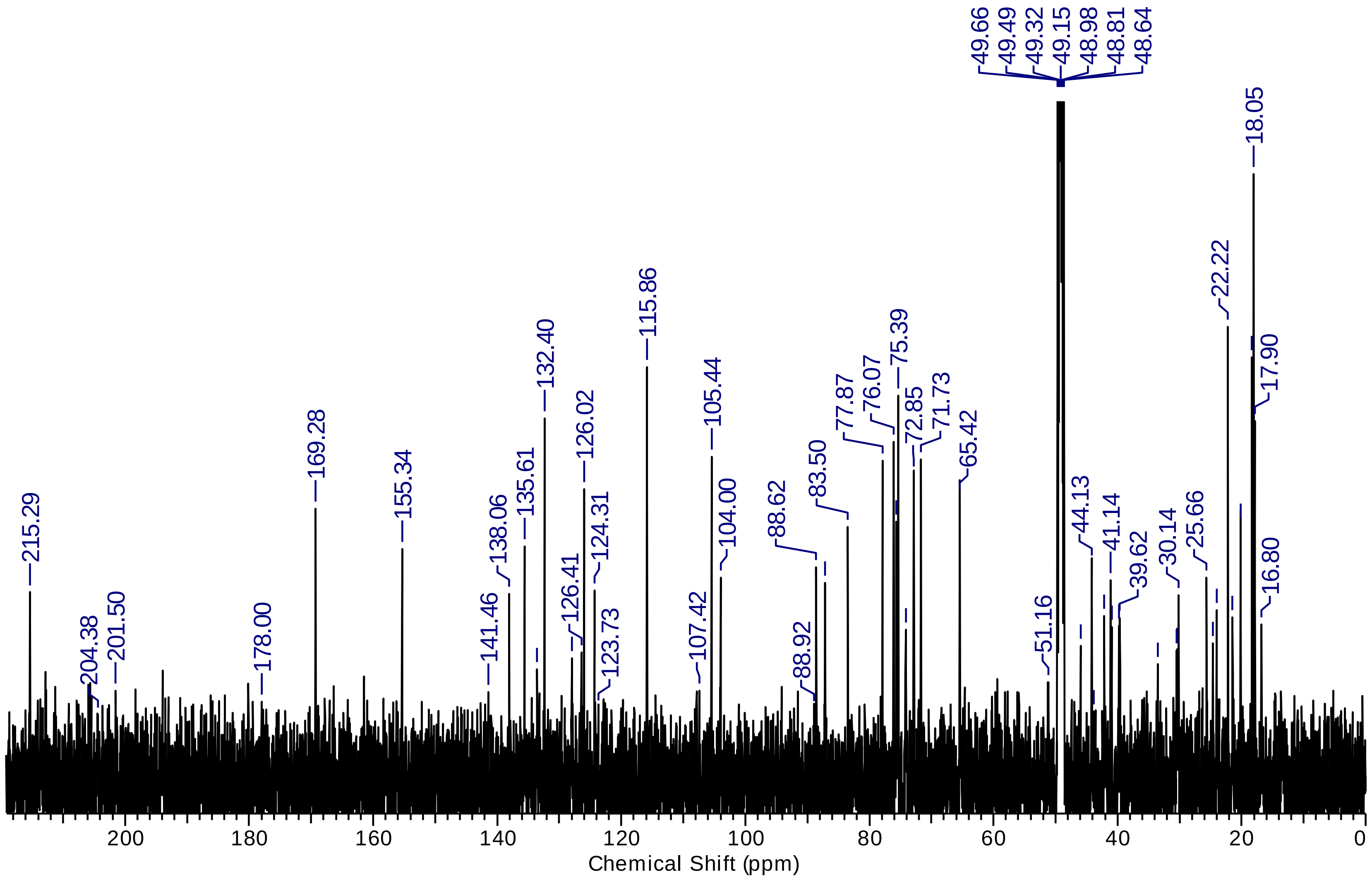

Figure S6. ^{13}C-NMR (125 MHz, methanol-d_4) spectrum of phocoenamicin B (**1**).

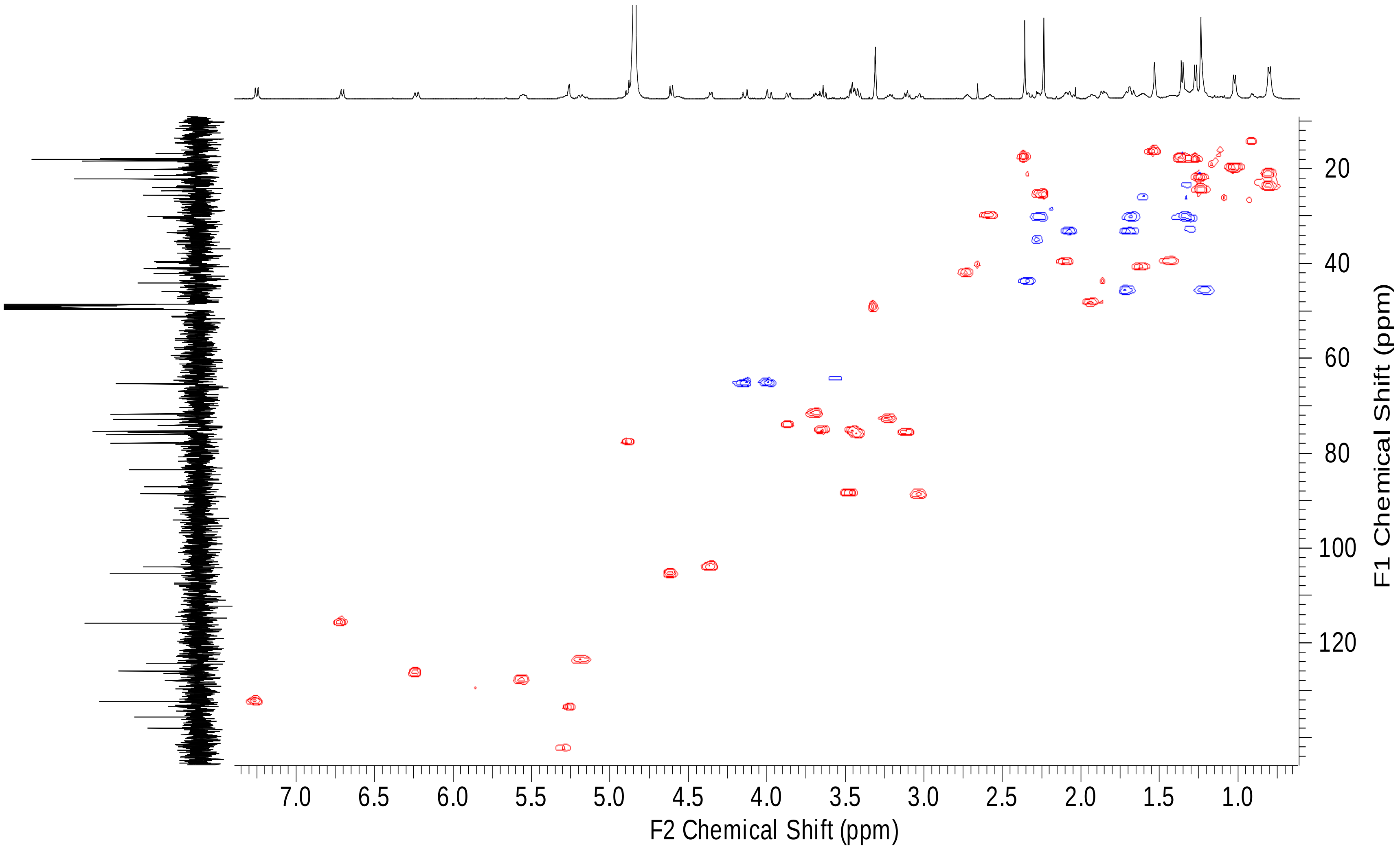

Figure S7. HSQC (methanol-*d*$_4$) spectrum of phocoenamicin B (**1**).

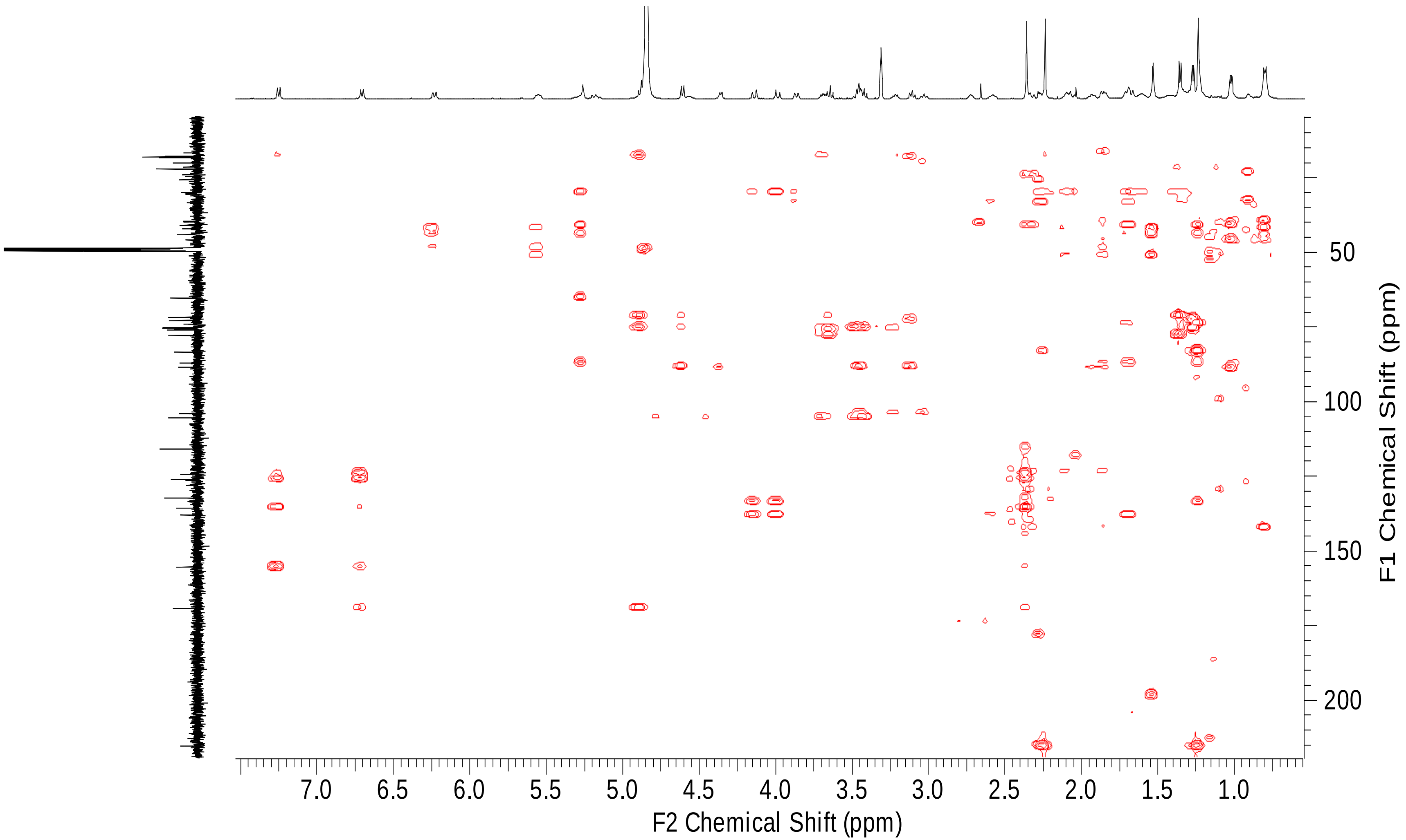

Figure S8. HMBC (methanol-d_4) spectrum of phocoenamicin B (**1**).

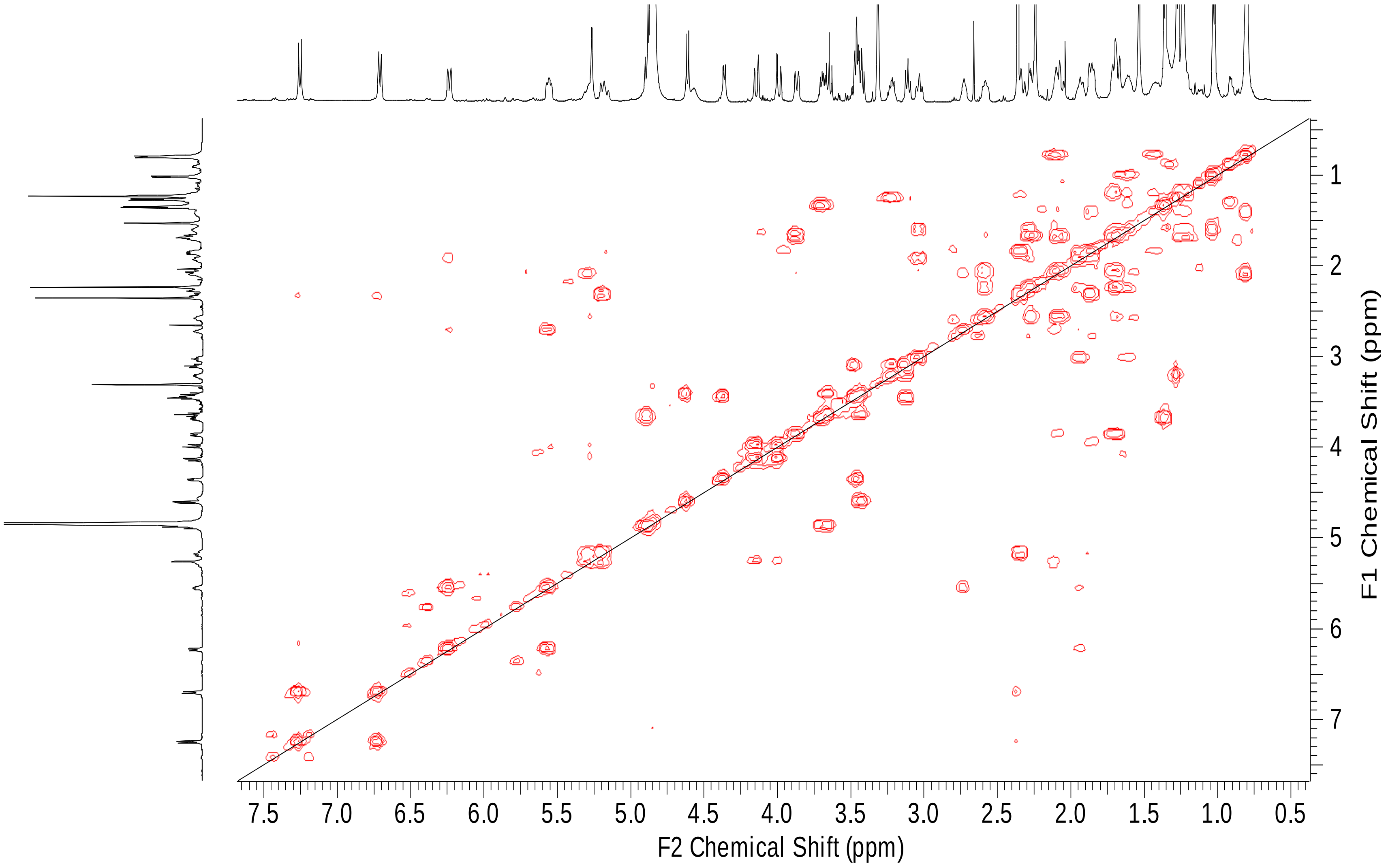

Figure S9. COSY (methanol-d_4) spectrum of phocoenamicin B (**1**).

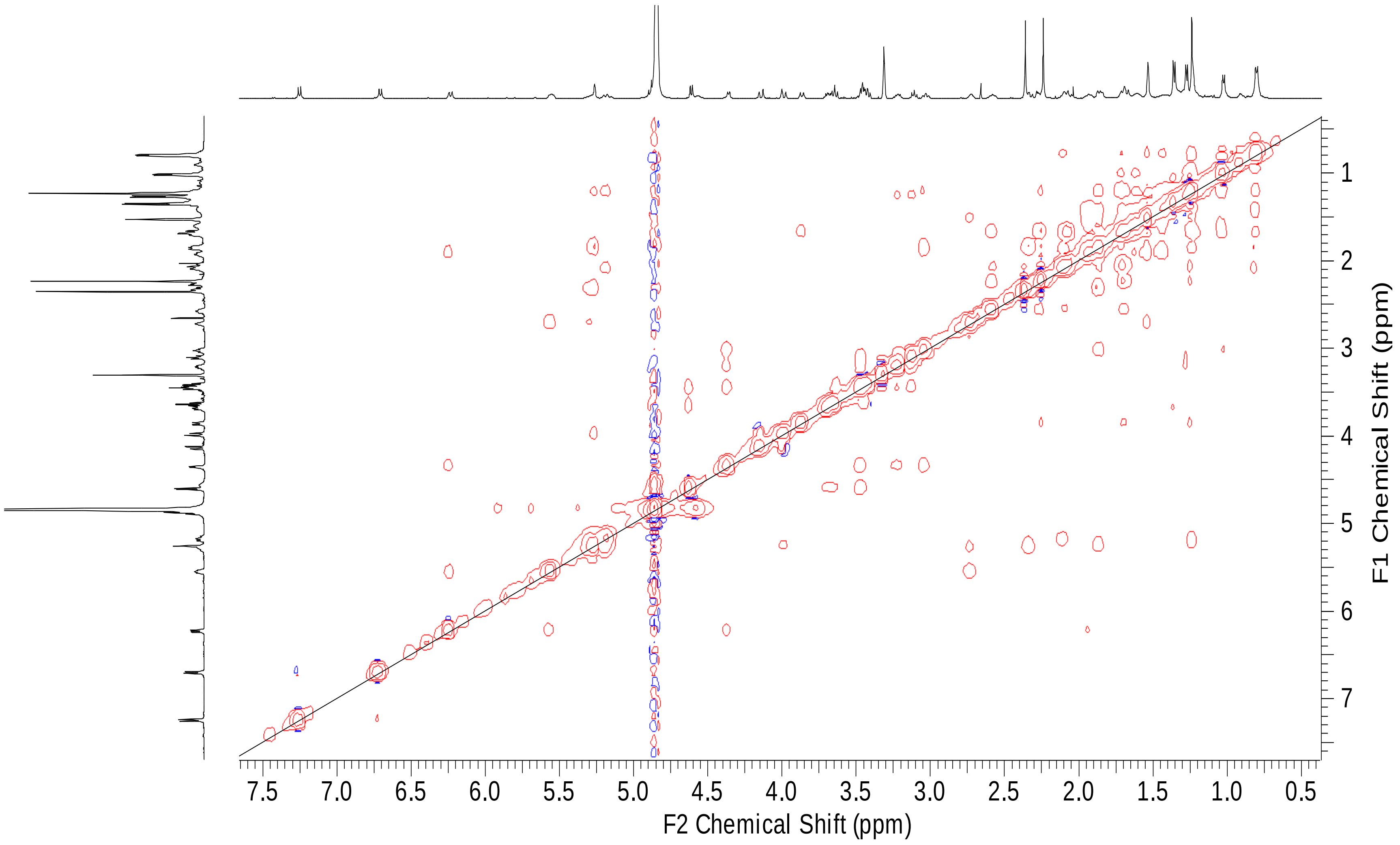

Figure S10. NOESY (methanol-d_4) spectrum of phocoenamicin B (**1**).

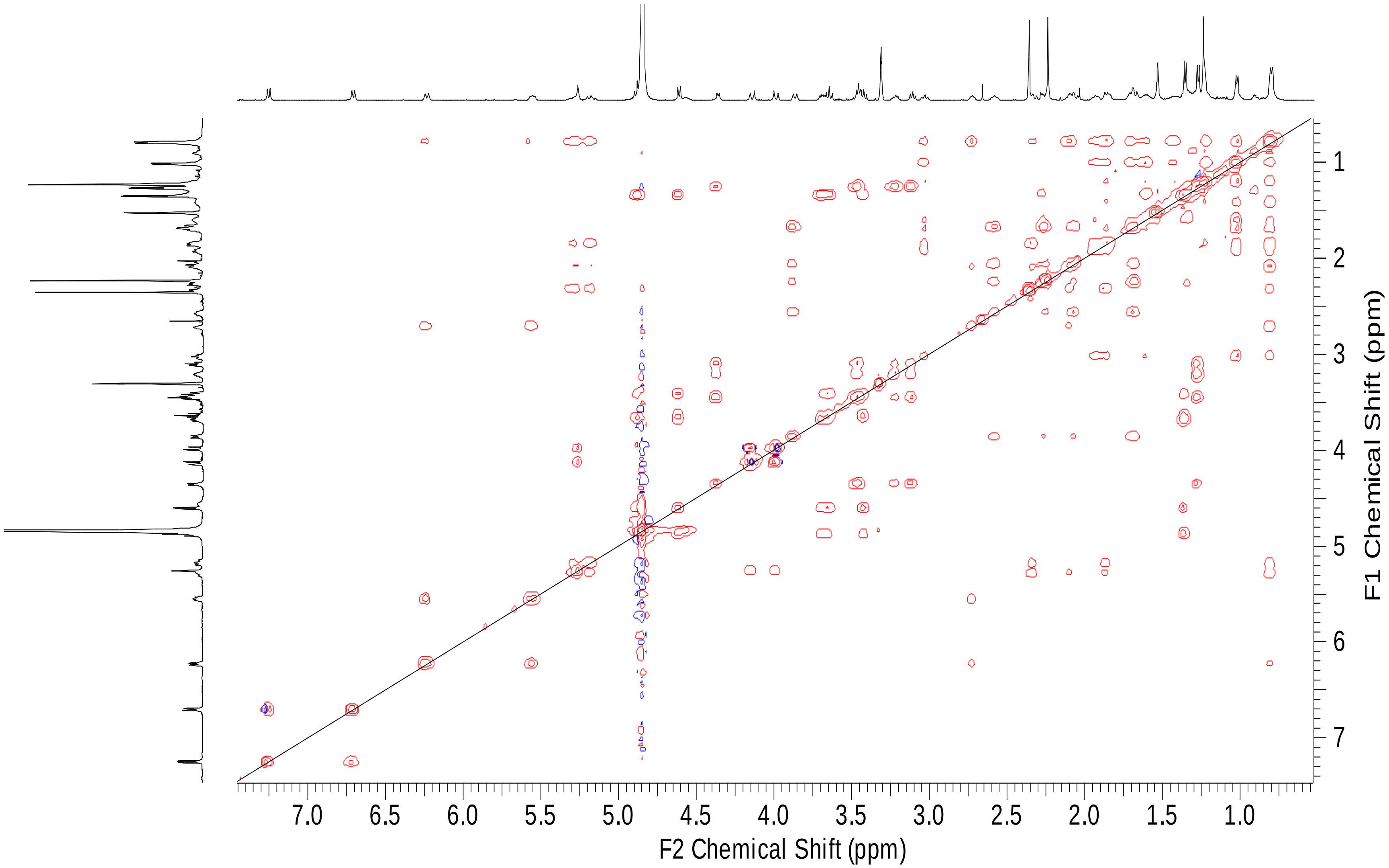

Figure S11. TOCSY (methanol-d_4) spectrum of phocoenamicin B (**1**).

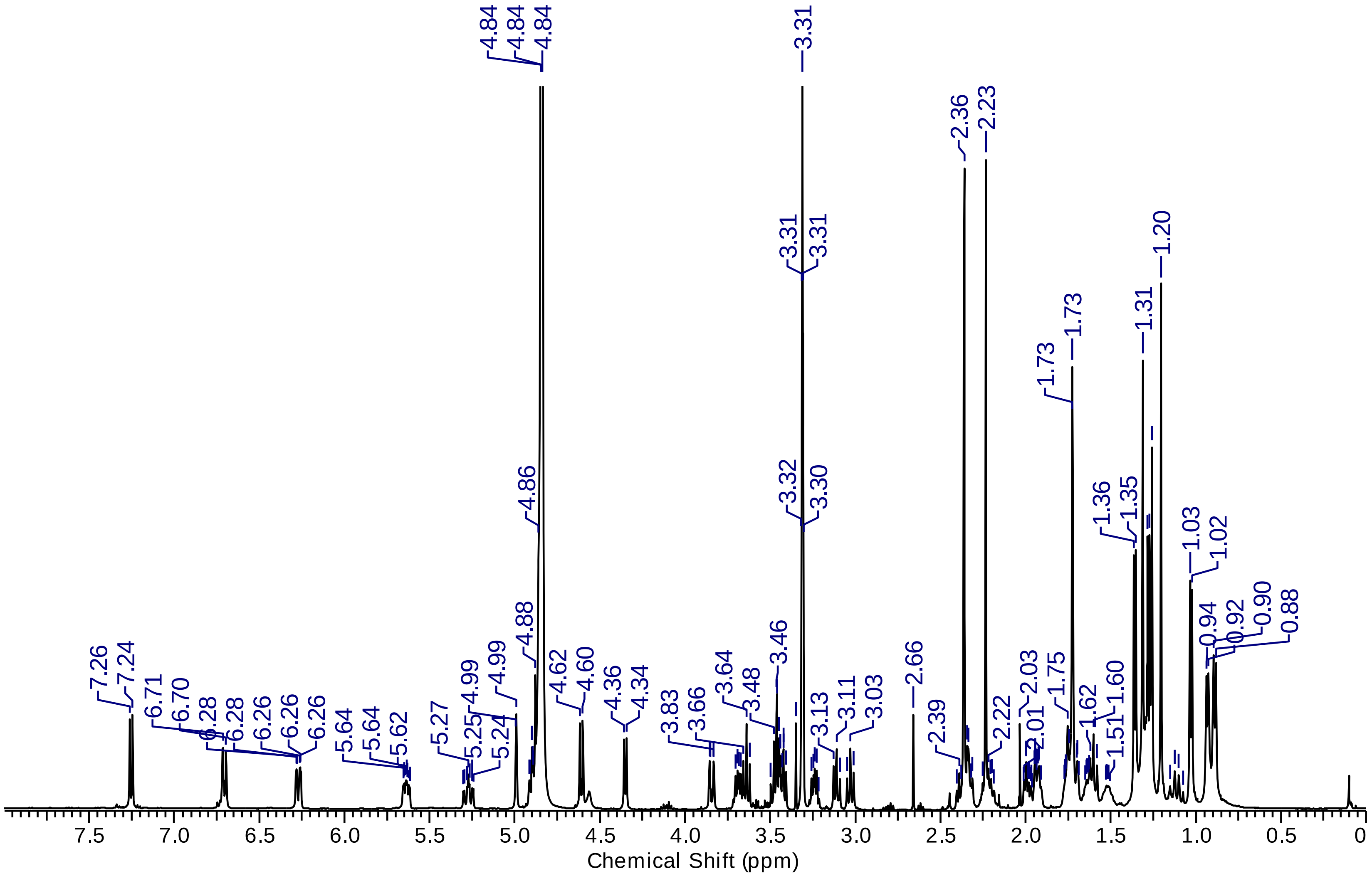

Figure S12. ^{1}H-NMR (500 MHz, methanol-d_4) spectrum of phocoenamicin C (**2**).

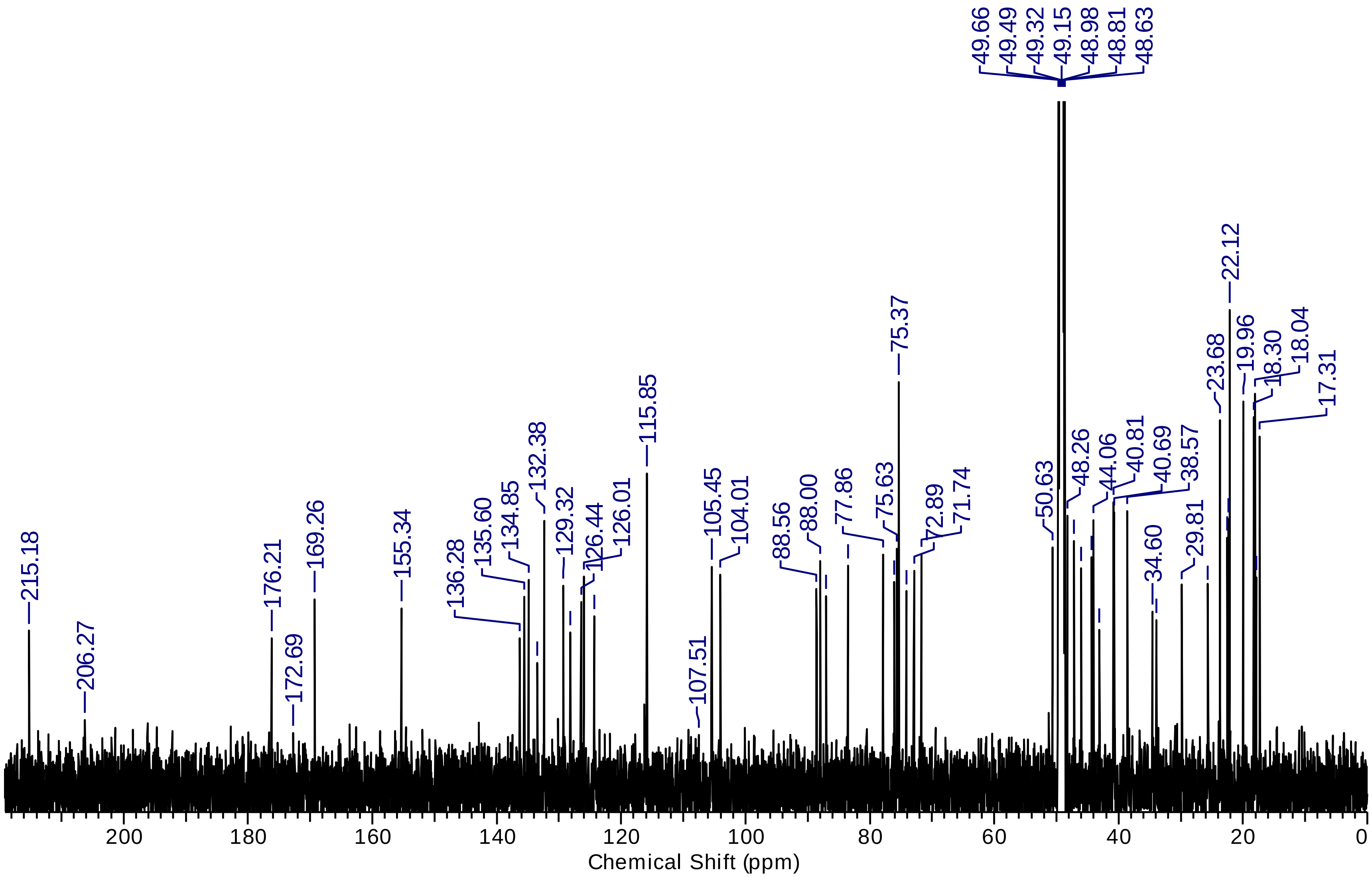

Figure S13. ^{13}C-NMR (125 MHz, methanol-d_4) spectrum of phocoenamicin C (**2**).

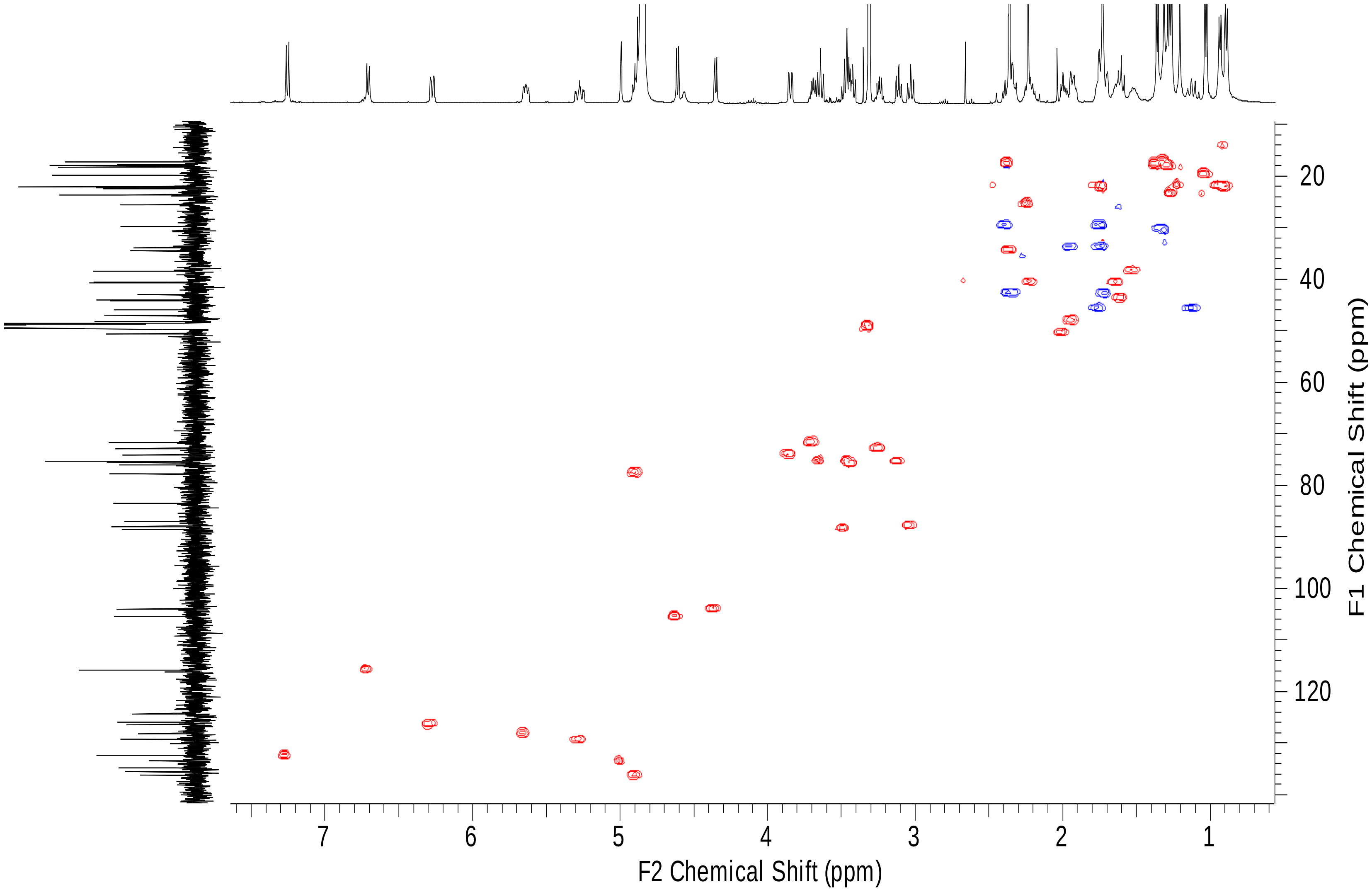

Figure S14. HSQC (methanol-d_4) spectrum of phocoenamicin C (**2**).

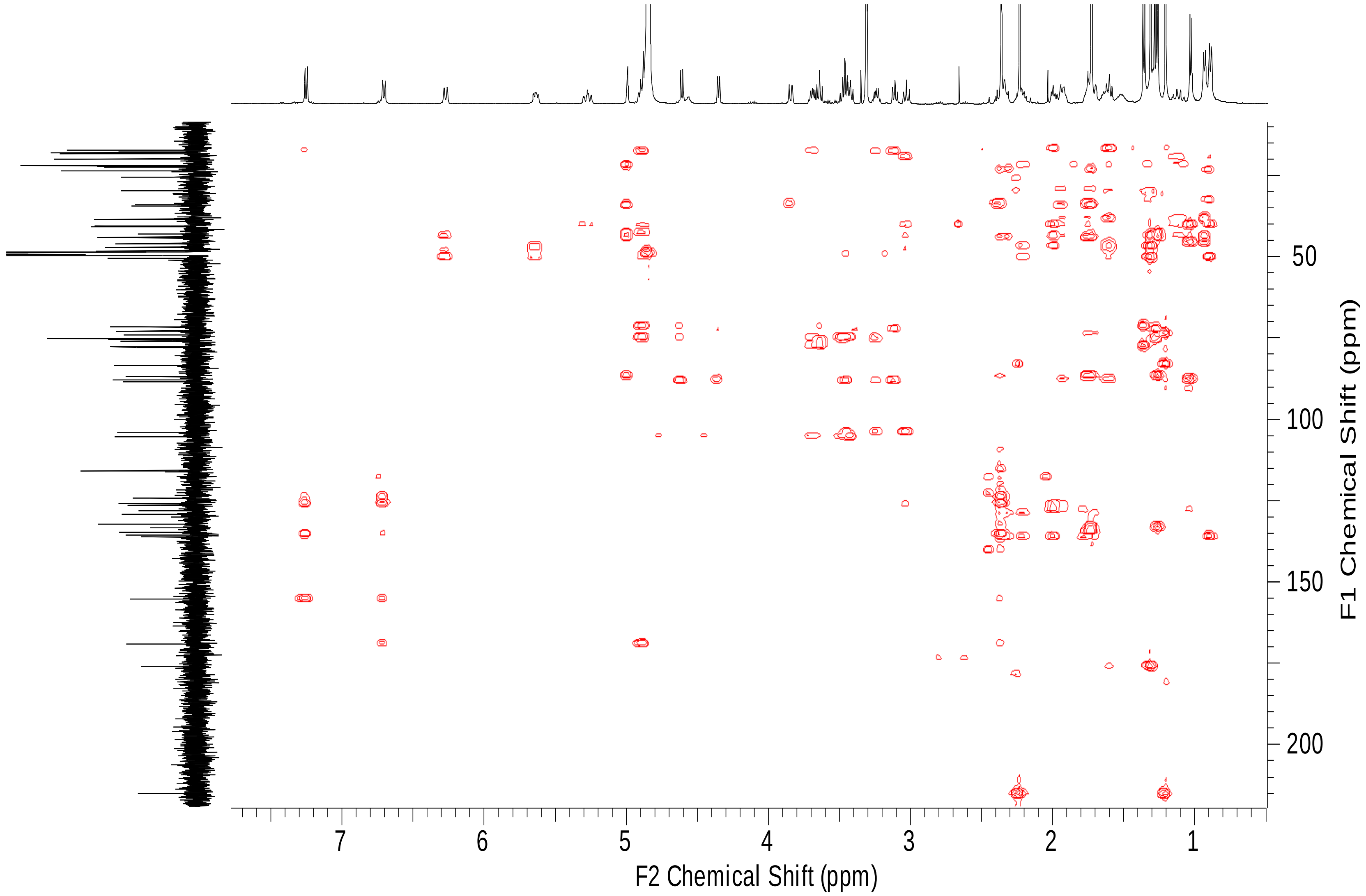

Figure S15. HMBC (methanol-d_4) spectrum of phocoenamicin C (**2**).

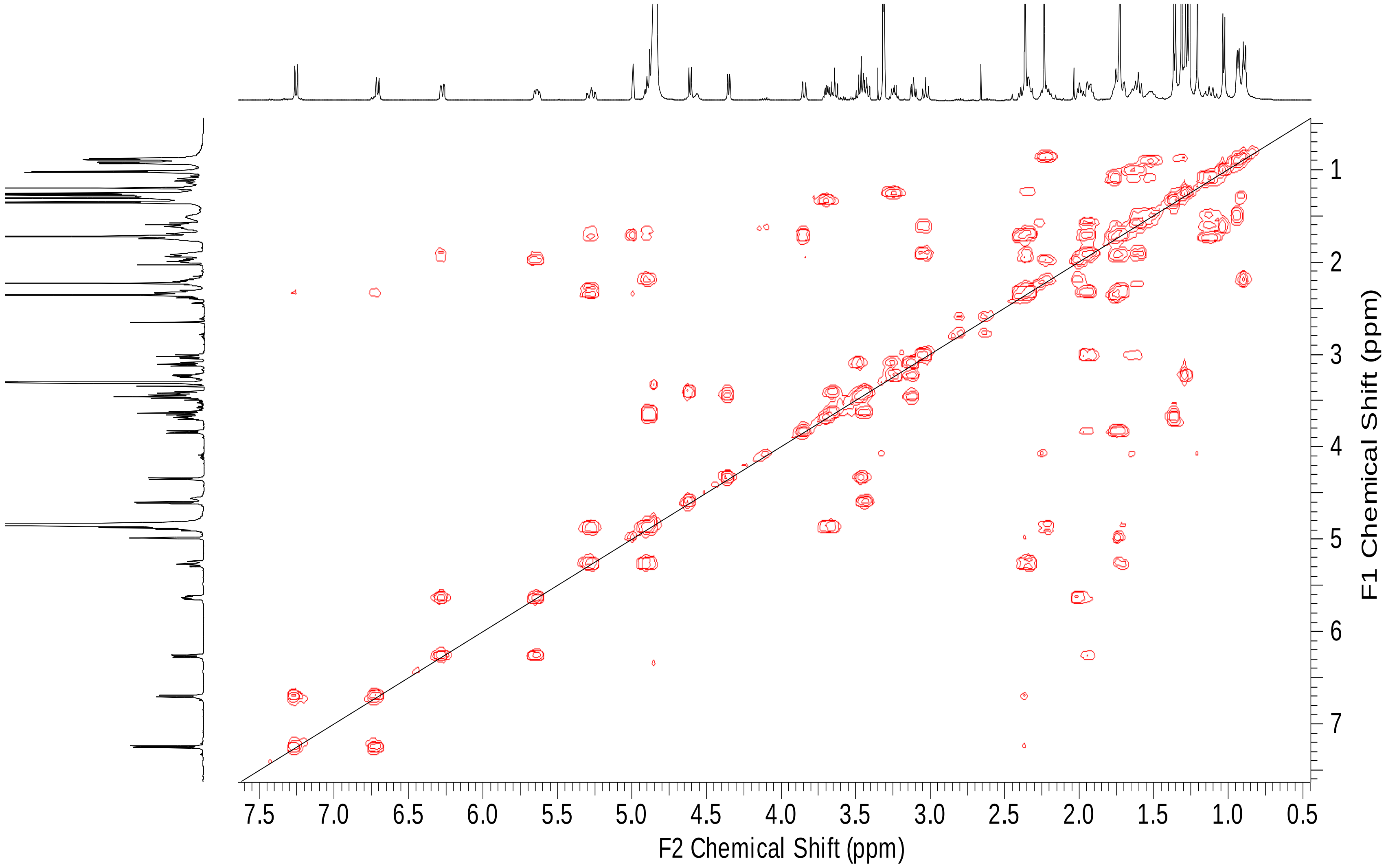

Figure S16. COSY (methanol-*d*$_4$) spectrum of phocoenamicin C (**2**).

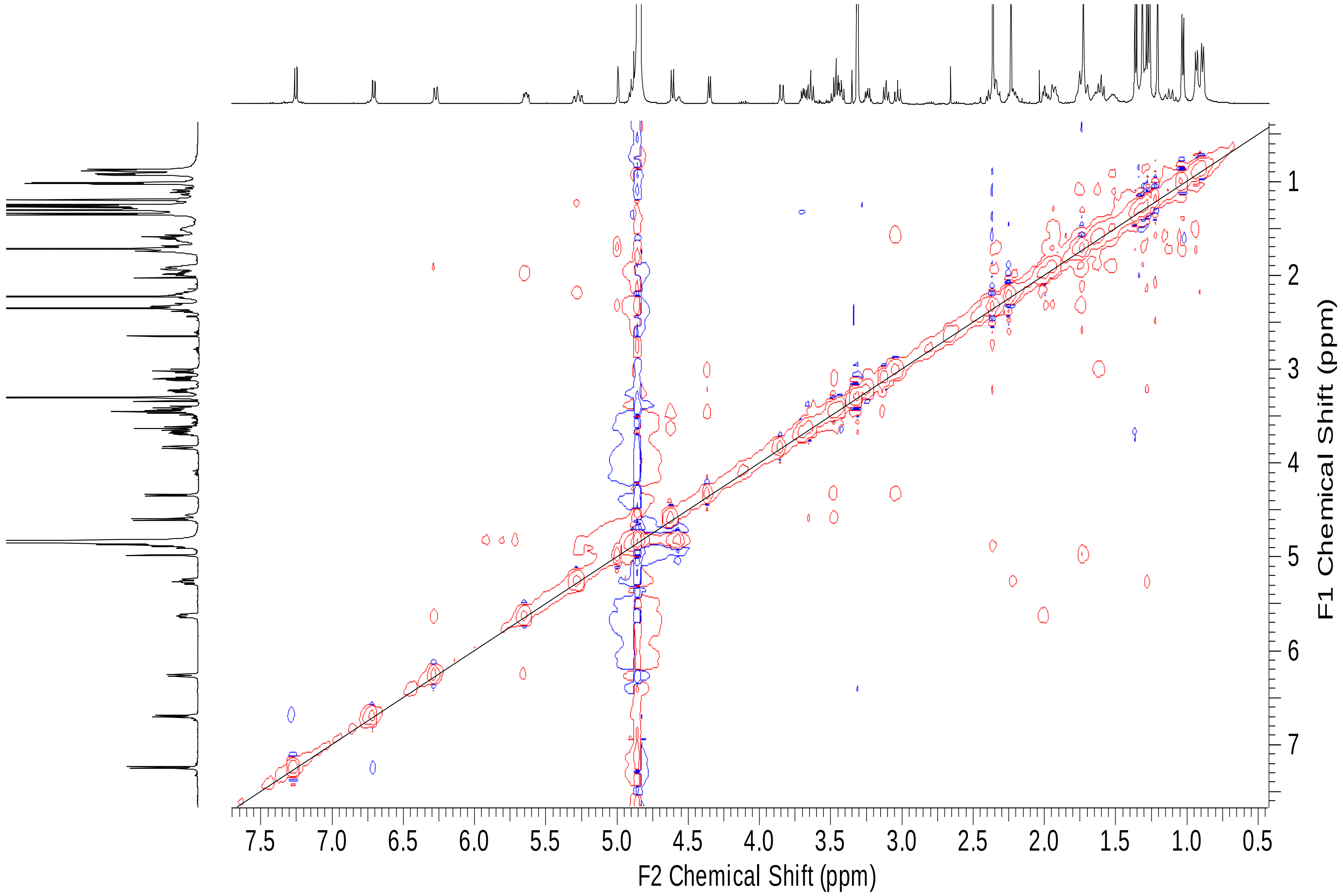

Figure S17. NOESY (methanol-d_4) spectrum of phocoenamicin C (**2**).

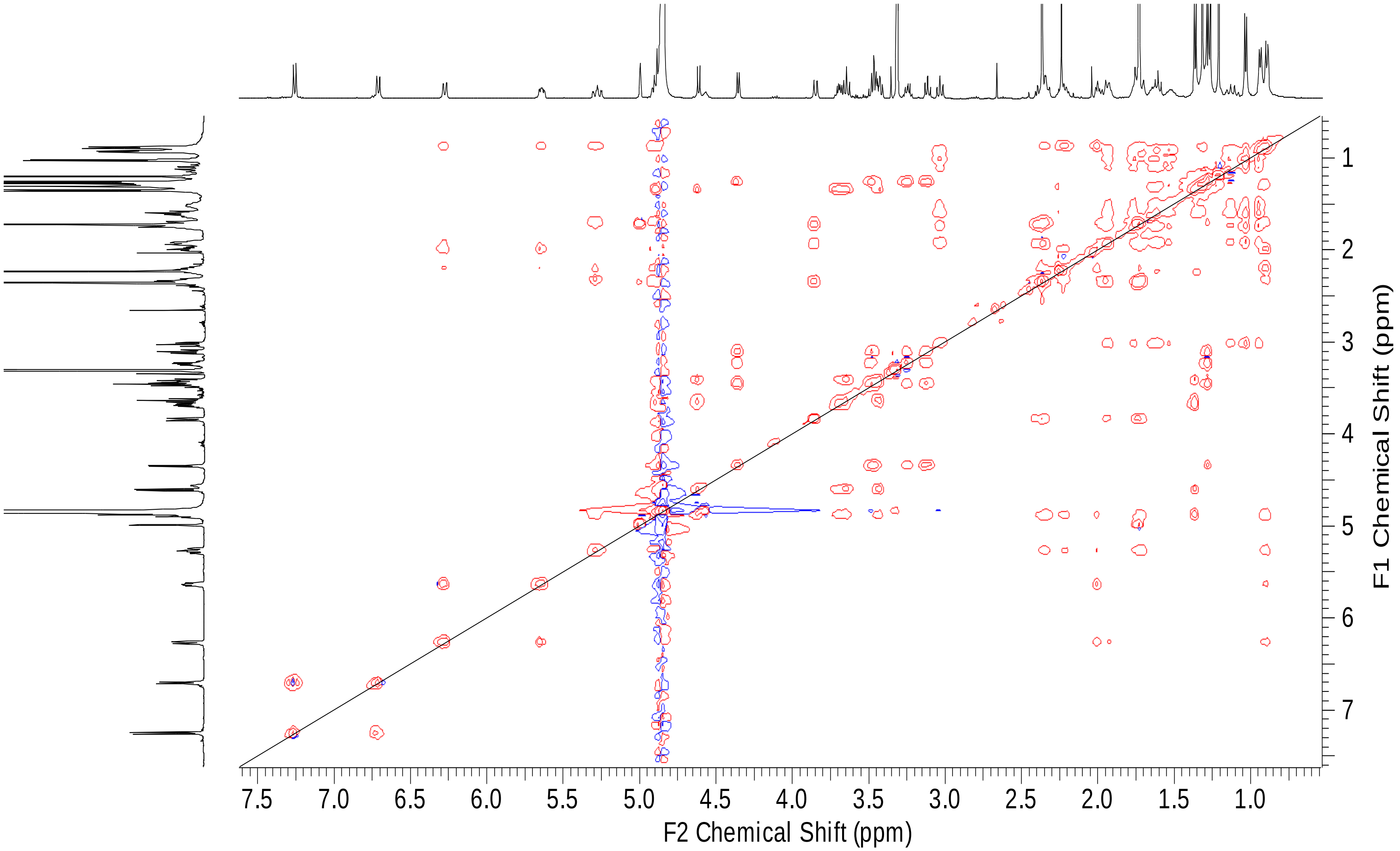

Figure S18. TOCSY (methanol-d_4) spectrum of phocoenamicin C (**2**).
